# Role of Valency and Defects in the Incorporation of Uranium into the Goethite [010] Surface: An Embedded Cluster Density Functional Theory Study

**DOI:** 10.1021/acsomega.5c00064

**Published:** 2025-04-23

**Authors:** Corinne H. Hatton, Angeliki Christodoulidou, Louise S. Natrajan, Nikolas Kaltsoyannis

**Affiliations:** Department of Chemistry, School of Natural Sciences, The University of Manchester, Oxford Road, Manchester M13 9PL, U.K.

## Abstract

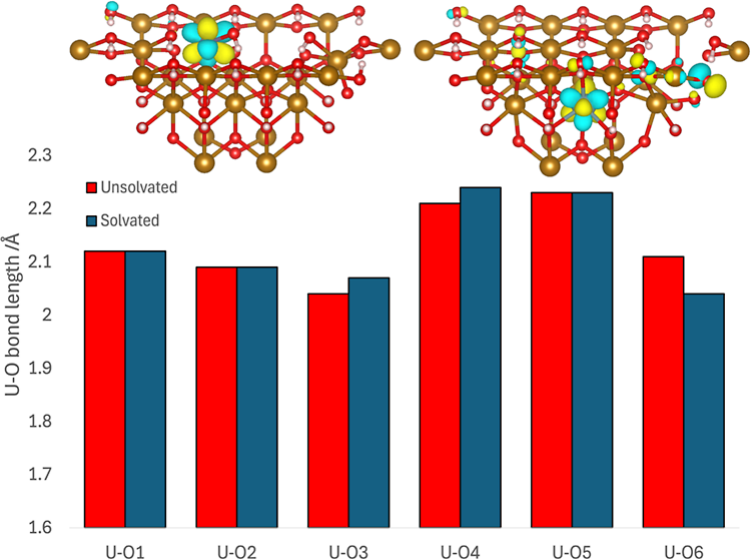

Incorporation of actinide species into iron (oxyhydr)oxides could present an environmentally secure method for preventing the release of actinides over an extended period, as would be the case in a number of radioactively contaminated land situations including surface, near-surface, and subsurface disposal and storage. Uranium is known to incorporate into iron (oxyhydr)oxides, including goethite, in a number of valence states, but the atomistic structures of these processes are unclear. In particular, it is increasingly reported that iron-containing minerals can reductively incorporate and stabilize the +V state of uranium, an oxidation state that is known to be unstable with respect to disproportionation. Here, we use density functional theory within the Periodic Electrostatic Embedded Cluster Method to model U(IV), U(V), and U(VI) incorporation into the pristine and iron-vacancy [010] surface and near-surface region of goethite. Solvated and unsolvated surfaces are studied, and the role of electron transfer from the lattice to uranium ions is explored. Comparisons are made with published X-ray absorption spectroscopic data, and we conclude that, based on the expected conditions for surface and near surface storage sites, both U(VI) and U(V) would incorporate into goethite as it transforms from ferrihydrite, forming two distinct structural types. We find that U(VI) incorporated into goethite may be reduced to U(V), where electron transfer occurs from oxygens surrounding iron vacancies and the incorporated uranium, reducing the U(VI) species to U(V). Both U(VI) and U(V) can incorporate into the surface of goethite with an adjacent iron vacancy, or U(V) can uniquely incorporate into the structure within the near-surface region, containing local but not immediately adjacent iron vacancies for charge compensation. Both of these incorporation schemes are little affected by the presence of a monolayer of surface water, suggesting that incorporation into goethite is a viable method to prevent uranium release into the aqueous surroundings.

## Introduction

Spent nuclear fuel poses a toxic and radiological hazard to both biological health and the environment and must therefore be disposed of securely and safely.^1−3^ Subsurface storage sites, including Geological Disposal Facilities (GDFs), have been identified as potential solutions for radioactive waste disposal in many countries, including the USA, UK, Finland, France, and Japan. The GDF concept utilizes multiple barriers, both engineered and natural, to isolate and contain radioactive wastes from spent nuclear fuel even in situations where the first levels of containment are breached.^1−4^ A thorough understanding of the complex (geo)chemistry of the components of the waste, particularly the key long-lived and heat generating actinides, including uranium, is important to inform decisions regarding long-term migratory behavior, the materials to be used in subsurface storage, and its physical location. Sorption processes between key elements in waste and components of the subsurface storage could be crucial to minimizing the release of radioactive materials.^2,3^ Understanding these interactions, whether natural or engineered, is an important factor in building confidence in radioactively contaminated land management especially when considering the very long time scales involved in the decay of elements contained within the waste.^1−4^

Uranium is a major component of spent nuclear fuel with complex chemical behavior, and generally its mobility is dependent on the oxidation state.^4^ In natural environments, uranium exists primarily in oxidation states +IV or +VI; while U(IV) is relatively immobile, U(VI) is more soluble and therefore mobile, typically existing as UO_2_^2+^ in aerobic environments.^5^ U(V) is less commonly observed, generally thought to be a short-lived, transient oxidation state, rapidly undergoing disproportionation, especially in the dioxo form UO_2_^+^.^4,5^ It has been demonstrated that U(V) is typically stable only in a narrow acidic or alkaline pH range.^6−9^ Recent research has shown the stabilization of the nonoxo pentavalent form of uranium U(V) in the presence of iron bearing minerals including green rust, hematite, and goethite under reducing conditions relevant to subsurface disposal including a GDF,^4,5,10−17^ and more remarkably, pentavalent U has been identified in a 1.6 billion year old sample of hematite.^18^ In the case of goethite, the incorporation mechanism is suggested to occur via electron transfer from Fe(II) to U(VI) sorped to ferrihydrite surface via an inner–sphere complex, as the crystallization process to goethite proceeds.^19^ Reduction from U(VI) in UO_2_^2+^ to U(V) and stabilization of U(V) could provide a means of minimizing uranium transport into the environment, yet the precise mechanisms of reductive incorporation of uranium remain undetermined.

Iron (oxyhydr)oxides are expected to be found in and around a GDF and in the subsurface through the corrosion of stainless steel and due to their high natural abundance in soil.^1,4^ Previous research has examined the interactions between iron oxides and actinide species, and a variety of sorption processes has been observed, including adsorption and absorption.^3,4,10−16,18−27^ Through the transformation of metastable iron (oxyhydr)oxides, adsorbed uranium can become incorporated into the mineral structure; a common example is the transformation of ferrihydrite to either hematite or goethite, the most stable form of iron (oxyhydr)oxide.^4,10−16,18,20−22^ Based on the likely conditions within subsurface storage sites and GDFs, and on previous experimental work, it is expected that goethite will preferentially form instead of hematite.^10,11^ It is believed that uranium may be incorporated into the goethite unit cell either through ferrihydrite transformation in soil or through the corrosion of stainless-steel barriers within a GDF. Incorporation of uranium into iron (oxyhydr)oxides has also been shown to yield long-lived products, preventing the release of uranium into the environment while also stabilizing the U(V) species.^4,10,12,13,15,18,24,27^ Reduction/oxidation of actinides by iron oxides is believed to be due to electron transfer between the redox-active actinides, such as uranium, and Fe(II)/Fe(III) whose redox potentials are coupled.^11^ Structural incorporation of uranium into iron (oxyhydr)oxides results in mineral phases that are more resistant to oxidation than uranium adsorption processes, thereby limiting remobilization into the surrounding environment.^10^ As such, incorporation is often seen to lead to reduced leaching rates and higher stability in a variety of conditions compared to adsorption.^1,10^ By contrast, uranium(IV) incorporation has been found only through the reduction of U(VI) species in highly reducing conditions that are unlikely to be found within a GDF.^14^

Incorporation of uranium species into goethite has been previously studied experimentally using Extended X-ray Absorption Fine Structure (EXAFS) spectroscopy; it was suggested that the distance of iron vacancies from incorporated uranium has a pronounced effect on the uranium oxidation state.^10,11^ Stagg et al. hypothesized that U(VI) could be incorporated into the near-surface region, corner-sharing to an iron vacancy.^10^ It was found that 52% of incorporated U(VI) was then reduced to U(V) through aqueous Fe(II) washing, and the U(V) incorporation was stable for 500 days.^10^ Massey et al.^11^ found that U(V) could also be incorporated into goethite; however, unlike Stagg et al., while iron vacancies were found, they were not necessarily within the local environment of the incorporated uranium, suggesting that iron vacancies function primarily as charge compensation sites.^11^

While these experimental studies show that uranium can incorporate into goethite as both U(VI) and U(V), the exact nature of the incorporation is unclear at an atomic level. Computational methods, however, can be used to gain this atomic-level insight, and in this study, embedded cluster Density Functional Theory (DFT) has been employed to assess a variety of models for the incorporation of uranium into goethite. Specifically, we study the incorporation of U(VI), U(V) and U(IV) into the goethite [010] surface and near-surface with and without the presence of nearby iron vacancies. The [010] surface is chosen as it has previously been found from both experimental and theoretical studies to be the most stable (i.e., it has the lowest surface energy) and is therefore the most abundant goethite surface cleavage crystallographic plane.^28−32^ In addition, there is only a singular OH termination of the [010] surface, simplifying its study.^32^ We also explore models in which explicit water molecules are added to the goethite surface to determine how solvation affects the interactions between uranium and the mineral structure.

## Methods Used and Systems Studied

DFT was employed in conjunction with the Periodic Electrostatic Embedded Cluster Method (PEECM),^33^ as implemented in the Turbomole code, version 7.3.^34^ This approach has been previously used to model short- and long-range surface adsorption interactions across a range of heavy metal systems.^35−39^ The PEECM consists of two key components: the point charge region and the embedded cluster, both based on the unit cell of the extended solid structure. The point charges represent the surrounding periodic environment interacting with the quantum mechanically treated embedded cluster, for which DFT is used.

**Figure 1:**
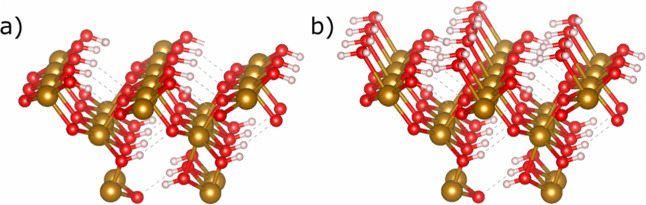
Embedded cluster of the [010] goethite surface from the unit cell of Yang et al.^40−42^ White spheres represent hydrogen atoms, red spheres represent oxygen atoms and gold spheres represent the iron centers. There are two different oxygen environments within the mineral structure, hydroxyl oxygens, and oxygens bridging between two iron atoms. Dashed lines represent hydrogen bonding. (a) unsolvated cluster containing 96 atoms. (b) Solvated cluster with 138 atoms, including 14 water molecules, one above each of the 14 surface Fe atoms.

A periodic supercell based on the experimental crystal structure at 273 K and 1 atm was produced from a goethite unit cell using the Crystal-17 and VESTA 3 codes.^40−42^ The supercell was then rotated and cut to produce a [010] surface slab with surface lattice parameters *a* = 4.5979 Å, *b* = 3.0178 Å, and *c* = 18.8200 Å. *a* and *b* were then used to generate the infinite 2D array of point charges, which is aperiodic in the *c* direction, 18.8200 Å being equivalent in depth to two goethite unit cells. Finally, the 96 atom, stoichiometric embedded cluster shown in Figure 1a, was cut out from the point charge slab and is slightly less than half the depth of the latter.

Within the PEECM, the quantum mechanically treated atoms at the edges of the embedded cluster, where it meets the point charge region, must be fixed in place to prevent significant geometric distortions on geometry optimization. The number of atoms in the center of the cluster that are allowed to relax during geometry optimization is then a balance between providing sufficient flexibility so as to accurately model the chosen chemistry and the computational cost of doing so. In the present work, two uranium incorporation positions within the embedded cluster were determined, and a study was conducted to determine how many atoms surrounding the incorporated uranium could be unfixed in place. At a minimum, the uranium, the oxygens surrounding it, and the iron vacancy positions were unfixed, 10 sites in total. Unfixing all the hydrogens surrounding the unfixed oxygens, plus additional three oxygens surrounding the separated iron vacancy, yields 24 unfixed sites. In between these extremes, trials to determine the optimum number of unfixed atoms were conducted, and on the basis of total energies, geometries, and spin densities, we determined that 17 unfixed atoms gave the best balance between computational speed and consistency of results.

The PBE0 hybrid density functional was used.^43^ The def2-SVP basis set was employed for iron, oxygen, and hydrogen,^44^ while for uranium, the Stuttgart-Köln Effective Core Potential (ECP) was used to describe the first 60 core electrons and was employed with the associated valence basis set.^45^ Spin densities and natural charges were obtained by using natural population analysis. The spin densities were used to determine the final oxidation state of uranium after electronic and geometric relaxation. A U(VI) species will have a spin density close to 0 as U(VI) does not possess any unpaired electrons. A U(V) species should have a spin density close to 1, and U(IV) close to 2, reflecting the U(V) [Rn]5f^1^ and U(IV) [Rn]5f^2^ electronic configurations. Mulliken population analysis was used to determine the contribution of individual atomic orbitals to molecular orbitals (MOs). All 3D representations of the optimized embedded cluster were imaged using VESTA 3, including views of the 5f-based MOs.^42^

The use of point charges based on formal oxidation states will likely lead to overpolarization of the electron density in the embedded cluster, and hence the values for the point charges were obtained using the iterative method described by Makkos et al.^35^ There are two different oxygen types in goethite, hydroxyl oxygens and bridging oxygens, and they have different natural charges. The natural charges used were Fe: +2.12, O: −1.18, O(H): −1.42 and H: +0.48.

To model solvation, 14 water molecules were added to the previously optimized surface, one above each surface Fe atom. Of these, two were placed above the section of the quantum mechanically treated region which was allowed to relax and hence were themselves geometry optimized. The remaining 12 were added with fixed geometries. Post optimization, all water molecules were altered to match the structure of the optimized waters, and the process was repeated until no further geometric changes occurred. The solvated model can be seen in Figure 1b.

**Figure 2:**
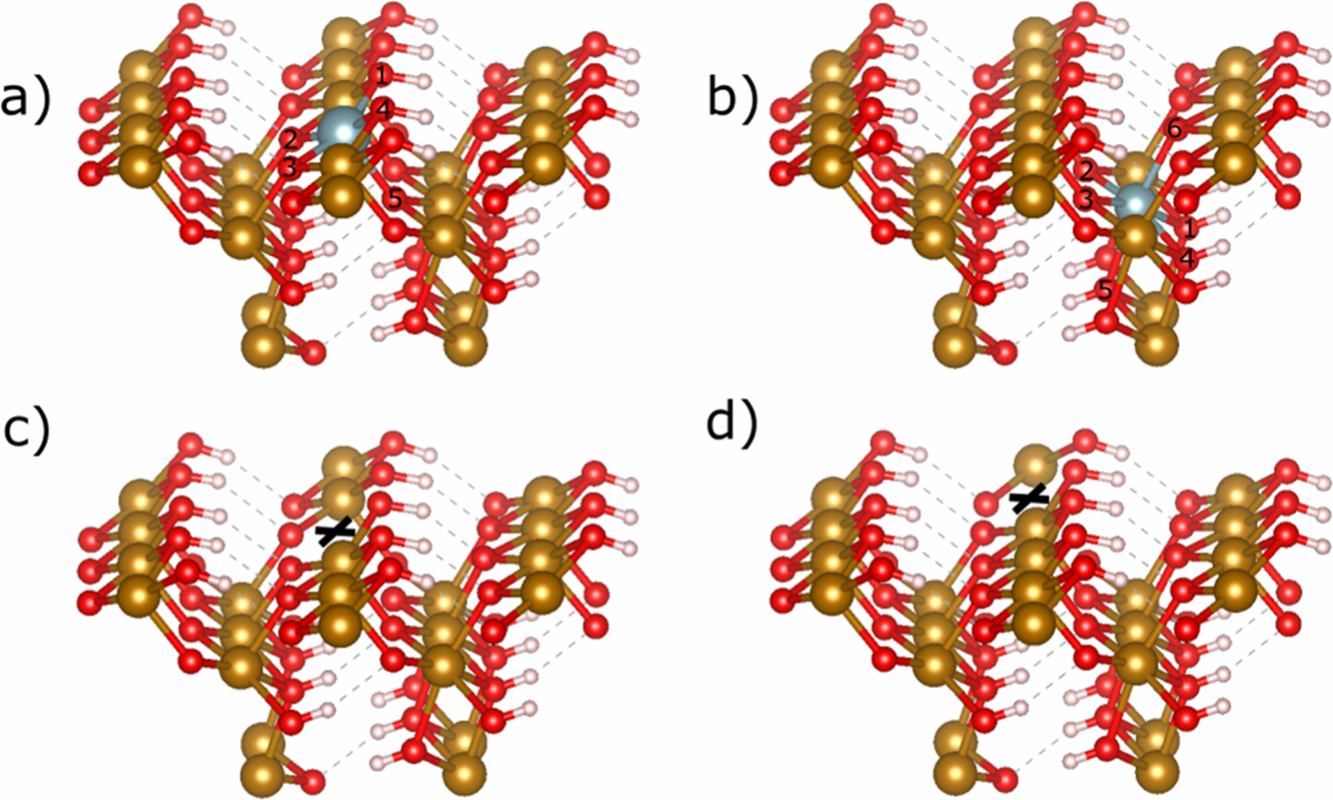
Uranium incorporation and iron vacancy positions within the goethite [010] surface embedded cluster. White spheres represent hydrogen atoms, red spheres represent oxygen atoms, gold spheres represent iron, and silver spheres represent uranium, which could be incorporated as U(IV), U(V), or U(VI). Iron vacancies are highlighted by a black cross, X. (a) Surface incorporation position of the uranium within a stoichiometric model of [010] goethite. (b) Near-surface incorporation position for uranium, within a stoichiometric model of [010] goethite. (c) Iron vacancy positioned adjacent to uranium incorporation sites, creating a non-stoichiometric model of [010] goethite. (d) Iron vacancy separated from uranium incorporation positions, creating a non-stoichiometric model of [010] goethite. Labeling of oxygens surrounding incorporated uranium for both surface and near-surface shown in (a,b), respectively. Oxygens 1, 4, and 5 are hydroxyl oxygens, while oxygens 2, 3, and 6 are bridging oxo-oxygens. For consistency, oxygen 1 is always the hydroxyl oxygen found closest to where iron vacancies are found and oxygen 2 is the closest oxo oxygen to an iron vacancy. When the surface is solvated, a sixth oxygen bond is seen in (a) between the incorporated uranium and the water on the surface. Dashed lines represent hydrogen bonding.

Uranium incorporation and iron vacancy positions were chosen based on the size and shape of the DFT region. Two different uranium incorporation positions were identified: surface and near-surface, as shown in Figure 2a,b. Two iron vacancy sites were also chosen; adjacent to the uranium positions, seen in Figure 2c, and separated from the uranium incorporation site, shown in Figure 2d. All of these models were also solvated as described above, resulting in 12 different incorporation schemes for each uranium oxidation state tested. Note that we consider the incorporation of only one uranium within the embedded cluster, as the concentrations of the uranium used in the experimental work are very low (a few hundred ppm), and no U–U interactions were found by EXAFS.^10,11^

All iron ions are Fe(III) and hence have five unpaired electrons. Previous studies have shown, both experimentally and computationally, that the high-spin Fe(III) configuration is the most stable.^46^ The uranium ions have up to two unpaired electrons. All of the embedded clusters are treated as high-spin. Goethite has complex magnetic behavior, but is believed to be predominantly antiferromagnetic at room temperature.^46−49^ However, modeling of the antiferromagnetic configuration of high-spin Fe(III) in goethite has previously been found to be challenging, significantly increasing the computational cost, with negligible changes in the optimized geometry with differing magnetic structures.^46,49^ Furthermore, previous modeling studies of goethite and actinide oxides have shown that the energy differences between ferromagnetic and antiferromagnetic ordering are small.^39,49^ We have therefore chosen to focus on the high-spin ferromagnetic arrangement, which is more computationally tractable than other spin solutions within an embedded cluster framework. We do not anticipate that the magnetic ordering will have any significant effect on geometry optimization, and as we focus on energy differences between incorporation models, it is very likely that such relative energies will be largely independent of magnetic ordering.

Table 1 provides the computational input details for each system studied. For the pure goethite [010] surface, 5 unpaired electrons were associated with each iron in the embedded cluster of 24 FeOOH units, giving a total of 120 unpaired electrons. For uranium incorporation models without an iron vacancy, one Fe(III) ion was removed from the cluster and replaced with a uranium ion, resulting in unpaired electron counts of 115–117 and overall charges of +3 to +1, depending on the uranium oxidation state. For models with an iron vacancy, two Fe(III) ions were removed from the cluster with one being replaced by a uranium ion, resulting in total unpaired electron counts of 110–112 and overall charges ranging from 0 to −2, again depending on the uranium oxidation state.

**Table 1:** Overall Charges and Total Number of Unpaired Electrons for the Models Used within This Work for Each Oxidation State of Incorporated Uranium

model/initial oxidation state	U(IV)	U(V)	U(VI)
uranium incorporated without an iron vacancy, with or without surface water	charge: +1	charge: +2	charge: +3
	total unpaired electrons: 117	total unpaired electrons: 116	total unpaired electrons: 115
uranium incorporated adjacent to an iron vacancy, with or without surface water	charge: −2	charge: −1	charge: 0
	total unpaired electrons: 112	total unpaired electrons: 111	total unpaired electrons: 110
uranium incorporated separated from an iron vacancy, with or without surface water	charge: −2	charge: −1	charge: 0
	total unpaired electrons: 112	total unpaired electrons: 111	total unpaired electrons: 110

Gibbs free energy corrections were applied to the self-consistent field energies by determining the harmonic vibrational frequencies within Turbomole and deriving the thermodynamic corrections using an in-house code, as described in Dempsey and Kaltsoyannis.^50^ These corrections to the electronic energy were determined using standard rigid-rotor harmonic oscillator model equations at conditions of 298.15 K and 0.1 MPa. Only the frequencies associated with the unfixed atoms were considered when calculating the Gibbs corrections.

## Results and Discussion

We have computationally probed a variety of models of the [010] goethite surface to explore the influence of iron vacancies on the oxidation state of incorporated uranium ions and the effects of solvation. The position of uranium relative to the iron vacancies and to the mineral surface was explored, as described in the Methods Used and Systems Studied section and illustrated in Figure 2. The initial uranium oxidation states tested were U(VI), U(V), and U(IV), although, as we shall see, the final oxidation state often varied from that specified on input. A full list of models studied is given in Supporting Information Section 8.

This section is ordered as follows. First, we present the incorporation of U(VI), U(V), and U(IV) cations within the [010] goethite surface with a variety of iron vacancy schemes, elaborating on how oxidation state, position relative to the surface, and iron vacancies affect relative stability. We then move on to evaluate the effect of water on these incorporation schemes, and finally, we compare our models to previous findings presented in the literature, highlighting which of our models are most similar to experimental data and thus build up a picture of how uranium in various oxidation states incorporates into surface and near-surface goethite [010].

### U(VI) Incorporation

U(VI) was found to remain as U(VI) only when adjacent to an iron vacancy, regardless of whether the uranium was placed in the surface or near-surface, as shown in Table 2 and in Supporting Information Table S7. In all other cases, electron transfer was found from goethite to uranium, resulting in the final oxidation state of U(V). The most favorable situation is when uranium is incorporated into the surface with an adjacent, edge-sharing iron vacancy. However, when the uranium is incorporated at the near-surface with a corner-sharing adjacent iron vacancy, a large energy increase is observed (see Supporting Information Table S7). This strong preference for surface incorporation of U(VI) species within goethite may well be due to the presence of two short, uranyl-like, bond lengths in the surface incorporation (1.82 and 1.91 Å) vs only one such, slightly longer distance in the near-surface (1.94 Å). For the U(VI) incorporation models with an adjacent iron vacancy, situations were also found where the U(VI) was reduced to U(V). A small change in the geometry of the incorporated uranium and the surrounding atoms was observed between these two models compared to when U(VI) remained as U(VI). The reduction of U(VI) to U(V) in both cases is less energetically favorable by ca. 50 kJ/mol than the analogous U(VI) cases. It may be that these U(VI) and U(V) surface incorporated cases are sufficiently close in energy that both situations could exist. As can be seen in Table 2, an elongation of the U–O distances was observed upon the reduction of U(VI) to U(V), with the average bond length for the surface incorporation with an adjacent vacancy increasing from 2.02 Å for U(VI) incorporation to 2.09 Å for U(V) incorporation through U(VI) reduction.

**Table 2:** Key U–O Bond Lengths (Å) in the Low-Energy Structures Obtained Following U(VI) and U(V) Incorporation into the Surface and Near-Surface of [010] Goethite, Using the Numbering Given in Figure 2. Spin Density on the U Also Shown and Used to Determine the Final Oxidation State^a^

U position	vacancy position		initial U oxidation state	U–O1	U–O2	U–O3	U–O4	U–O5	U–O6	average U–O	spin density of U	final oxidation state	relative energy
surface	adjacent	edge-sharing	U(VI)	1.81	1.91	2.06	2.17	2.14		2.02	0.12	U(VI)	0.0
surface	adjacent	edge-sharing	U(VI)	2.00	1.94	2.14	2.19	2.18		2.09	–1.05	U(V)	+58.3
near-surface	separated		U(VI)	2.12	2.09	2.04	2.21	2.23	2.11	2.13	–1.06	U(V)	+79.8
surface	adjacent	edge-sharing	U(V)	1.99	1.95	2.16	2.21	2.15		2.09	1.25	U(V)	+28.3
near-surface	separated		U(V)	2.12	2.06	2.06	2.23	2.24	2.11	2.14	1.22	U(V)	0.0

a Relative energies in kJ/mol. Note that the relative energies can be used only to compare models with the same initial oxidation state, as initial U(VI) models have a different number of electrons to models where U(V) was initially specified; full explanations can be found in the Supporting Information Section 1. Complete data set for U(VI) and U(V) incorporation into all models can be found in Supporting Information Section 2 Tables S7 and S8.

**Figure 3:**
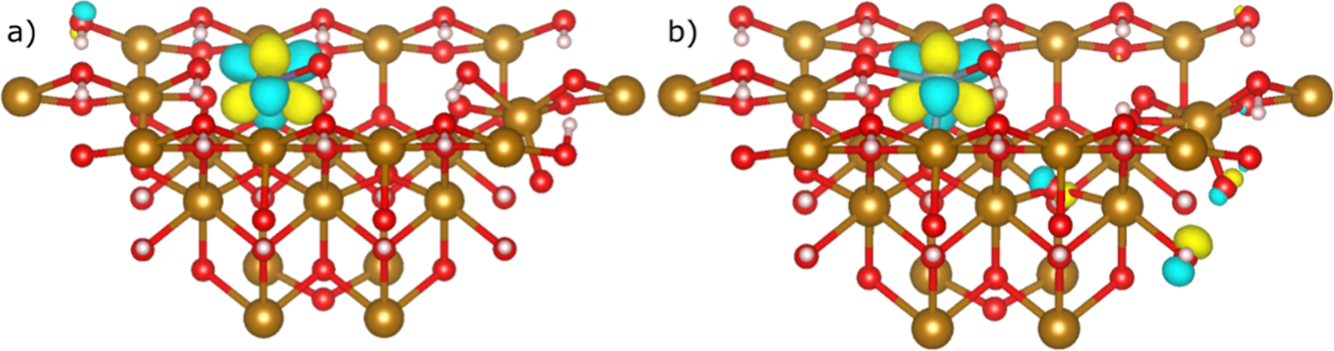
U 5f-based MOs for goethite [010] surface-incorporated uranium species with an adjacent iron vacancy. (a) Reduction of initially specified U(VI) to U(V), MO has 95% 5f character, (b) U(V) incorporation, MO has 81% 5f character. Isovalue = 0.05. White spheres represent hydrogen atoms, red spheres represent oxygen atoms, gold spheres represent iron, and silver spheres represent uranium. Orbital phases are indicated by yellow and blue. Hydrogen bonding is not shown for clarity.

Changes in the spin density and charge before and after uranium incorporation were then used to determine which atoms contribute to the electron transfer to the uranium. For both surface and near-surface incorporation with an adjacent iron vacancy, the reduction of U(VI) to U(V) was found to be a consequence of electron transfer from the under coordinated oxygens surrounding the iron vacancy and the oxygens bonded to the incorporated uranium. Full descriptions of changes in spin density and charge are gathered in Supporting Information Section 7 for all models investigated. To further probe the oxidation state of the incorporated uranium species, the 5f-based MOs were imaged, and their atomic orbital character was determined. For U(VI) incorporation, there should be no MOs with significant uranium 5f character due to U(VI) formally having no 5f valence electrons. For U(V) incorporation, a single 5f-based MO would be expected. To verify this, Figure 3 shows the 5f-based MOs for surface uranium incorporation with an adjacent vacancy in two scenarios, where U(VI) was reduced to U(V) vs when U(V) was specified on input. The results are similar, suggesting that U(VI) is indeed reduced to U(V) when there is an adjacent vacancy.

When U(VI) incorporation with a separated vacancy was attempted, the final state was consistently U(V), as shown in Table 2 and Supporting Information Table S7. There is some variation in the calculated U–O bond lengths between the models with a separated iron vacancy and those with an adjacent iron vacancy because of the position of the iron vacancy and flexibility of the goethite structure when there is an adjacent vacancy. These may result from the difference in rigidity and flexibility of the goethite mineral structure between the adjacent iron vacancy situation and with a separated iron vacancy. The reduction of U(VI) to U(V) in the separated vacancy cases comes from electron transfer mainly from the oxygens surrounding the incorporated uranium, with some also observed from under-coordinated oxygens surrounding the vacancy, as evidenced by changes in spin density and partial atomic charges. Greater electron donation comes from oxo oxygens than hydroxyl oxygens, by a factor of approximately two (full details can be found in Section 7 in the Supporting Information).

For the models without a vacancy, a similar situation was found, whereby U(V) was found to incorporate through the reduction of U(VI) (Supporting Information Section 7). Electron transfer occurs from the oxygens surrounding the incorporated uranium, with the oxo oxygens again donating nearly twice as much electron density as the hydroxyl oxygens (Supporting Information, Section 7). In addition to spin density and charge data, visualization of 5f-based MOs once again verified U(V) incorporation for both the separated iron vacancy and the no vacancy models. These images can be found in Supporting Information Section 6.

The relative energy of models with the same starting total number of electrons and overall charge can be compared to determine the energetic favorability of the various incorporation schemes. For the no vacancy models, surface incorporation is clearly much more favorable than near-surface incorporation, Supporting Information Table S7. In the presence of iron vacancies, the two lowest-energy cases arise from either U(VI) or U(V) incorporation at the surface with an adjacent vacancy; the latter is 58 kJ/mol less stable than the former. The next most stable incorporation, at +80 kJ/mol, is for U(V) at the near-surface with a separated iron vacancy. The remainder of the incorporation cases are significantly less stable, especially at the near surface with an adjacent vacancy. We conclude that U(VI) will incorporate only at the surface with an adjacent iron vacancy, whereas U(V) could be found either at the surface with an adjacent vacancy or at the near-surface with a separated iron vacancy.

### U(V) Incorporation

A similar incorporation study was then undertaken with the initially stipulated U(V). As discussed above, incorporation of initially stipulated U(VI) leads in most cases to reduction to U(V); here, as shown in Table 2, in all cases of initially stipulated U(V) incorporation, the uranium remains as U(V), suggesting that incorporated U(V) is a stable oxidation state, with no further actinide-mineral redox processes occurring. When comparing U(VI) reduction vs U(V) incorporation in Table 2, there is a small amount of variation of calculated bond lengths between models with the same uranium incorporation scheme with U(V); however, the average bond lengths calculated for each model remain similar. The relative energies are remarkably similar to those of the U(VI) incorporation models. Once again, the most energetically favorable situations are when U(V) is incorporated at the surface with an adjacent iron vacancy or at the near-surface with a separated iron vacancy; these two cases differ by only 28 kJ/mol. This is a sufficiently small gap, such that U(V) incorporation may occur at more than one type of site. When comparing to the U(VI) incorporation models, it should be borne in mind that these models differ in the total charge and the total number of unpaired electrons, and as such any minor changes in bond lengths and energy between U(VI) reduction to U(V) and direct U(V) incorporation are likely a consequence of the reduction process. Overall, though, we see that both the U(VI) and U(V) incorporation models produce very similar results through different routes.

### U(IV) Incorporation

**Figure 4:**
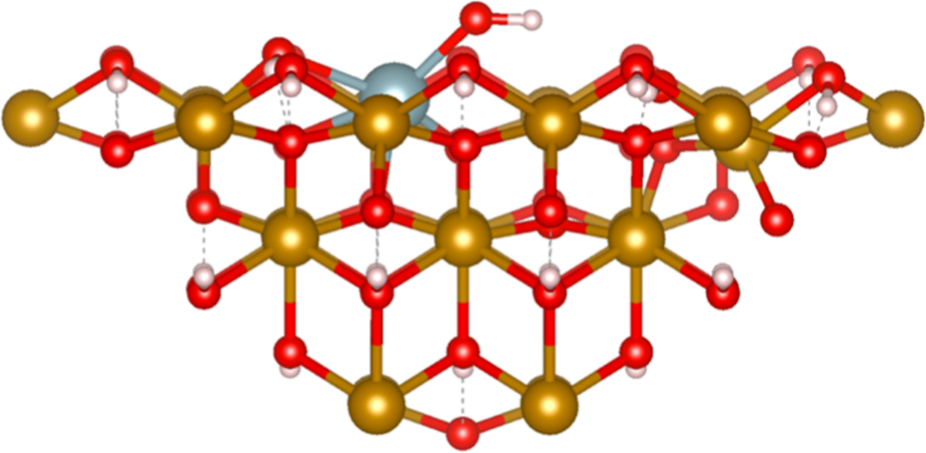
Incorporation of U(IV) into the surface of [010] goethite with an adjacent iron vacancy, showing distortion of the lattice. White spheres represent hydrogen atoms, red spheres represent oxygen atoms, and gold spheres represent iron. Dashed lines represent hydrogen bonding.

The incorporation of U(IV) within goethite has been seen experimentally,^10,14^ although this requires extremely reducing conditions and occurs through the reduction of U(VI) or U(V) after their incorporation, rather than via direct U(IV) incorporation. We did not see any reduction from U(VI) or U(V) to U(IV) in the calculations described in the previous sections but have explored direct U(IV) incorporation into the surface and near-surface in a variety of vacancy schemes (Supporting Information Table S9). Unlike U(VI), but as with U(V), in these situations, no actinide-mineral redox processes are observed, with only U(IV) final states located. The relative energy difference between U(IV) models has the same trend as for U(V) incorporation (Supporting Information Table S9) where near-surface incorporation with an adjacent vacancy is the most energetically favorable. At both the surface and near-surface, U(IV) incorporation causes elongation of U–O bonds compared to U(V) and U(VI). For the surface incorporation with an adjacent iron vacancy, an average U–O bond length of 2.20 Å was calculated, compared to 2.02 and 2.09 Å for U(VI) and U(V), respectively. This is believed to be a consequence of the increase in the uranium ionic radius with a decreasing oxidation state and the lack of any short uranyl-like U–O bond lengths calculated for U(IV) incorporation. In addition, U(IV) incorporation results in a significant distortion of the surrounding lattice in all incorporation schemes, as illustrated in Figure 4, for surface incorporation in the presence of an adjacent iron vacancy.

### Solvation Effects

**Figure 5:**
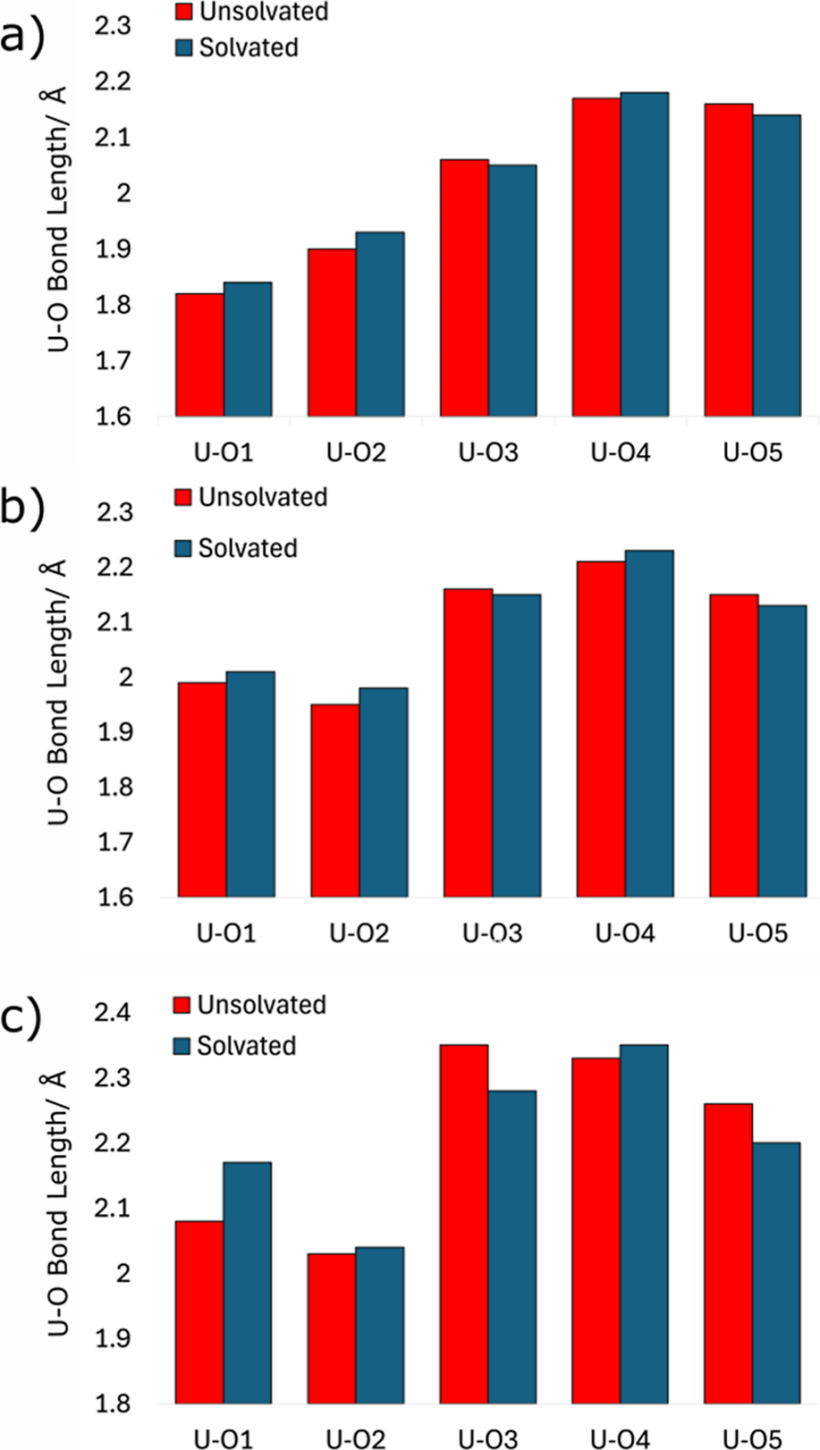
Comparison of U–O bond lengths pre- and postsolvation for (a) U(VI), (b) U(V), and (c) U(IV) incorporation into the [010] goethite surface with an adjacent iron vacancy. The complete set of bond lengths can be found in Supporting Information Section 2.

It is important to consider the effects of solvation on the incorporation of uranium into goethite to better understand what may occur should a GDF be breached by groundwater. Thus, all of the models discussed above were solvated with a layer of explicitly treated water molecules. In general, we find that adding a layer of water molecules has little effect on the computed U–O distances, as is illustrated in Figure 5, which plots the U–O distances in analogous unsolvated and solvated systems with adjacent iron vacancies. Similar plots were constructed for the separated iron vacancy and no vacancy models (Supporting Information Section 3), and solvation was again seen not to have a significant effect on the calculated uranium–oxygen bond lengths for any oxidation state. Furthermore, in systems for which the unsolvated calculations find no change in the U-oxidation state on goethite incorporation, adding a layer of surface water gives the same result. In the situations where U(VI) is reduced to U(V), analysis of the electronic structure shows that the electronic transfer to the U does not involve the waters.

Adding a layer of explicit water molecules does, however, affect the relative energies between incorporation models, as shown in Table 3. For U(VI), in the absence of water, incorporation at the surface with an adjacent vacancy is the most energetically favorable model, whereas for U(V), it is the incorporation at the near-surface with an adjacent vacancy. However, after solvation, for both U(VI) and U(V), surface incorporation with an adjacent iron vacancy is the most favorable. For U(IV), the most favorable model after solvation is the same as without water; near-surface incorporation with a separated vacancy. Upon solvation, the energy difference to reduce U(VI) to U(V) in the adjacent vacancy models does not change significantly: 58 kJ/mol vs 67 kJ/mol pre and post solvation at the surface. For the near-surface, this reduction changes from 49 to 45 kJ/mol (see Supporting Information Tables S7 and S10). Solvation generally reduces the energy difference between the most favorable models, for example, U(V) incorporation into the near-surface with a separated vacancy. For U(VI) reduction to U(V) in this model, presolvation at a relative energy of +80 kJ/mol was found compared with the surface U(VI) incorporated with an adjacent vacancy. Post solvation, this relative energy difference drops to only 31 kJ/mol. This suggests that if uranium incorporation into goethite occurs in the presence of water, a wider variety of incorporation schemes may be seen. More specifically, over time, exposure to water may result in a higher proportion of incorporated U(VI) and U(V) being located at the surface rather than the near-surface.

**Table 3:** Relative Energies (kJ/mol) between Different Solvated Uranium Incorporation Models with Either a Single Iron Vacancy or without an Iron Vacancy^a^

	U(IV)	U(V)	U(VI)
U at surface, adjacent vacancy	+13.8	0	0 + 66.5
U at surface, separated vacancy	+135.8	+145.7	+217.2
U at near-surface, separated vacancy	0	+40.7	+30.7

a Full description of relative energy calculations given in Supporting Information Section 1. For U(VI) incorporation with an adjacent vacancy, two relative energy values are given. The first is where U(VI) is incorporated as U(VI) and the second is where U(VI) is reduced to U(V). For all U(VI) incorporation models where there is a separated vacancy or no iron vacancy, the final oxidation state of the incorporated uranium species is U(V).

### Comparison with Experiment

Uranium incorporation into goethite has been previously studied experimentally, and the results have differed in their predicted incorporation models.^10,11^ Based on the data obtained herein, three models have been identified that best match experimental bond length data, as shown in Table 4. These models are also the lower energy ones, further suggesting that they are the most likely.

**Table 4:** Comparison of Experimental U–O Bond Lengths (Å) with the Best Fit Computational Models Generated in This Study^a^

experimental data/computational model	uranium final valency	U–O1	U–O2	U–O3	U–O4	U–O5	U–O6	U–O7	average U–O bond length
Stagg et al.^10^	U(VI)	1.82 (0.8)	2.03 (0.8)	2.23 (2.2)	2.42 (2.2)				2.22
Stagg et al.^10^	U(V)	2.17 (5.2)	2.45 (1.8)						2.24
Massey et al.^11^	U(V)	2.18 (5.5)							2.18
solvated surface uranium with an adjacent vacancy	U(VI)	1.84	1.93	2.05	2.18	2.14		2.51	2.11
solvated surface uranium with an adjacent vacancy	U(V)	2.01	1.95	2.14	2.22	2.18		2.51	2.17
solvated near-surface uranium with a separated vacancy	U(V)	2.12	2.06	2.09	2.26	2.23	2.04		2.13

a For the experimental data, the numbering of U–O bonds does not match the numbering given in Figure 2 and (b), instead it represents the path type designation derived from EXAFS data. Additionally, the average bond length data are determined based on the coordination number, which is indicated in brackets. For models within this study, the numbering follows the same scheme as Figure 2 and (b). U–O7 specifically refers to the interaction between surface uranium and the closest water molecule from the solvated surface. To calculate the average of our models, all six calculated U–O bonds were averaged.

Our models which are closest to the data found by Stagg et al.^10^ are those with U(VI) and U(V) incorporated at the solvated surface with an adjacent vacancy. The calculated U–O bond lengths from the solvated models more closely match the EXAFS-derived equivalents than unsolvated models, on account of the long U–O bond lengths found experimentally, 2.42 and 2.45 Å. These distances are typical of uranium–water interactions. We find a short, uranyl-like bond length only when U(VI) is incorporated into the surface with an adjacent vacancy, and this matches Stagg et al.‘s data closely, 1.82 vs 1.84 Å. For U(V) incorporation, an average U–O bond length of 2.17 Å was calculated, which matches the 2.17 Å U–O bond length with a coordination number of 5.2, as found by Stagg et al. One discrepancy between our models and the work by Stagg et al. is the specific geometry of the uranium relative to the iron vacancy. Stagg et al. found that uranium species, as either U(VI) or U(V), incorporate edge-sharing to an iron vacancy; however, we find that similar bond lengths are found when uranium species are incorporated at the corner-sharing position. Our models and the experimental results both incorporate uranium species at the surface, suggesting that the position of the uranium relative to the surface is more important than whether it is edge- or corner-sharing to the iron vacancy, as these are a consequence of the crystal plane.

For near-surface incorporation, we find that our model with a separated vacancy most closely matches the experimental data provided by Massey et al.^10^ The latter give the average U–O bond length as 2.18 Å, whereas in our study, we suggest a bond average of 2.13 Å, with a range from 2.04 to 2.26 Å.

Taking the relative energies from Table 3 together with the previous experimental conclusions, we can form an overall picture of uranium incorporation. Surface U(VI) incorporation with an adjacent vacancy is the most favorable, closely followed by U(V) incorporation at the near-surface with a local, nonadjacent iron vacancy with a relative energy of either +31 kJ/mol (U(V) produced via initial U(VI) incorporation) or +42 kJ/mol (direct U(V) incorporation). The other likely incorporation scheme is U(V) at the surface with an adjacent iron vacancy, which is either formed through the reduction of U(VI) or through the direct incorporation of U(V). Based on all these results, we suggest that uranium incorporates in two structural positions: at the surface with an adjacent vacancy and at the near-surface with a separated vacancy, as either U(VI) at the surface or U(V) for both the surface and near-surface positions. The distribution of these incorporation schemes will likely depend on the exact conditions found with the GDF. This is further evidenced by the different experimental results seen by Stagg et al. and Massey et al.^10,11^

To summarize, this study has examined a variety of uranium incorporation schemes into the goethite [010] surface, probing the effect of actinide oxidation state, incorporation position, the presence/location of iron vacancies, and the effects of surface solvation. For the first time, the PEECM has been used for studying metal ion incorporation into a mineral surface, finding that electron transfer from undercoordinated oxygen ions is responsible for the reduction of U(VI) cations to U(V) into goethite. Using bond length data, relative energies, and comparison with experimental results, it is proposed that within subsurface and GDF conditions, both U(VI) and U(V) would incorporate into goethite as it transforms from ferrihydrite, forming two distinct structural types: both U(VI) and U(V) can incorporate into the surface of goethite with an adjacent iron vacancy or U(V) can incorporate into the structure within the near-surface region, containing local but not immediately adjacent iron vacancies for charge compensation.

## Environmental Implications

When spent fuel and nuclear waste storage facilities are designed, it is important to assess the ability of ubiquitous iron-bearing minerals such as goethite to hinder the release of radioactive elements into the environment. Using DFT within the PEECM, models have been developed for U(IV), U(V), and U(VI) incorporation into the pristine and iron-vacancy [010] surface and near-surface region of goethite. It is observed that both U(VI) and U(V) will likely incorporate into one of the three incorporation schemes: U(VI)/U(V) surface incorporation with an adjacent iron vacancy and U(V) within the near-surface with a nonadjacent iron vacancy. U(VI) is found only when it is incorporated next to an adjacent iron vacancy; more commonly, U(VI) is reduced to the less mobile U(V) oxidation state. We propose that all three of these incorporation schemes will exist simultaneously due to the small energy differences between them, although the exact distribution will depend upon environmental conditions. For U(IV), likely incorporated into goethite only under conditions unlikely to be found in a GDF, large distortion of the lattice is seen. Surface solvation was found to have only minor effects on the interactions between incorporated uranium species and the mineral structure, and hence, uranium ions may well remain within the goethite lattice even in the presence of water. Overall, our findings that U(VI) and U(V) can incorporate into goethite, both in and without the presence of surface water, suggest that uranium species will be immobilized, preventing their release into the environment if breached by groundwater.

## Supporting Information Available

Relative energy descriptions, complete set of unsolvated and solvated data, calculated frequency data, 5f MO images, changes in spin density compared to equivalent unincorporated [010] goethite, and complete list of models made with images (PDF)
