# Supplementary material for: Role of Valency and Defects in the Incorporation of Uranium into the Goethite [010] Surface: An Embedded Cluster Density Functional Theory Study
Source: ACS Omega. 2025 Apr 23;10(17):17717–26. doi: 10.1021/acsomega.5c00064 (PMC12059891; doi:10.1021/acsomega.5c00064)
Supplement: Supplementary file 1 — ao5c00064_si_001.pdf [file ao5c00064_si_001.pdf]

Role of Valency and Defects in the Incorporation of Uranium into the Goethite [010] Surface: An Embedded Cluster Density Functional Theory Study

Corinne H. Hatton, Angeliki Christodoulidou, Louise S. Natrajan, and Nikolas Kaltsoyannis[*]

Department of Chemistry, School of Natural Sciences, The University of Manchester, Oxford Road, Manchester M13 9PL, UK

* Corresponding author's email: nikolas.kaltsoyannis@manchester.ac.uk

Pages: 117

Figures: 71

Tables: 36

Supporting Information

This document provides supporting information for this paper, including descriptions for the determination of the relative energies between models, all calculated U-O bond lengths for all models made, frequency data, and images of 5f-based molecular orbitals.

Coordinate files are located in an accompanying Mendeley database. Other files included there are example input files and output files. The database can be found as: Hatton, Corinne (2024), “Uranium Incorporation into [010] goethite input files”, Mendeley Data, V1, doi: 10.17632/c7wczbh8w7.1

Table of Contents

Section 1: Relative energy descriptions and explanations ***.......... 3***

Section 2: Complete set of unsolvated incorporation data ***.......... 15***

Section 3: Solvation data ***.......... 19***

Section 4: Calculated Frequency data ***.......... 23***

Section 5: Bar charts comparing effect of solvation on calculated U-O bond lengths ***.......... 68***

Section 6: 5f MO imaging ***.......... 77***

Section 7: Changes in spin density compared to equivalent unincorporated [010] goethite model ***.......... 89***

Section 8: List and images of models made ***.......... 111***

Section 1: Relative energy descriptions and explanations

Note that only models of the same initial oxidation state and number of iron vacancies can be compared with one another, as comparisons can be made only between systems with the same total number of electrons.

The SCF energy from the geometry-optimised model was noted in Hartrees. Then for each model the vibrational frequencies were calculated, and Gibbs corrections calculated, as per Dempsey et al.[1] The lowest energy model in the groups being compared was then identified and set to a reference energy of 0 Hartrees. The relative energy differences are then converted into kJ/mol. The aim of this is to identify and compare equivalent structures to determine the most stable structural configurations for each oxidation state. The results of this method can be seen in Tables S1-6.

Table S1: Comparative energy calculations for U(VI) incorporation into [010] goethite.

Model	Final oxidation of U	E in Hartree	Gibbs correction to E in Hartree	New E in Hartree	Relative E in Hartree	Relative E in kJ/mol
Surface U with an adjacent iron vacancy	U(VI)	-31897.10359	0.02036	-31897.12395	0.00000	0.0
Near-surface U with an adjacent iron vacancy	U(VI)	-31896.78592	0.02531	-31896.81123	0.31272	+821.0
Surface U with an adjacent iron vacancy	U(V)	-31897.07622	0.02552	-31897.10174	0.02221	+58.3
Near-surface U with an	U(V)	-31896.77121	0.02123	-31896.79244	0.33151	+870.4

adjacent iron vacancy						
Surface U with a separated iron vacancy	U(V)	-31897.02072	0.01705	-31897.03777	0.08618	+226.3
Near-surface U with a separated iron vacancy	U(V)	-31897.07999	0.01358	-31897.09357	0.03038	+79.8
Surface U with no iron vacancy	U(V)	-33159.40924	0.02369	-33159.43293	0.00000	0.0
Near-surface U with no iron vacancy	U(V)	-33159.23116	0.02380	-33159.25495	0.17798	+467.3

Table S2: Comparative energy calculations for U(V) incorporation into [010] goethite.

Model	E in Hartree	Gibbs correction to E in Hartree	New E in Hartree	Relative E in Hartree	Relative E in kJ/mol
Surface U with an adjacent iron vacancy	-31897.31782	0.01652	-31897.34434	0.01079	+28.3
Near-surface U with an adjacent iron vacancy	-31897.02435	0.01447	-31897.03882	0.31632	+830.5
Surface U with a separated iron vacancy	-31897.27006	0.02168	-31897.29174	0.06334	+166.4
Near-surface U with a separated iron vacancy	-31897.33311	0.02202	-31897.35513	0.00000	0.0
Surface U with no iron vacancy	-33159.92443	0.02393	-33159.94836	0.00000	0.0

Near-surface U with no iron vacancy	-33159.71762	0.01319	-33159.73119	0.21755	+571.2

Table S3: Comparative energy calculations for U(IV) incorporation into [010] goethite.

Model	E in Hartree	Gibbs correction to E in Hartree	New E in Hartree	Relative E in Hartree	Relative E in kJ/mol
Surface U with an adjacent iron vacancy	-31897.41739	0.01700	-31897.43439	0.02540	+66.6
Near-surface U with an adjacent iron vacancy	-31897.10999	0.01937	-31897.12936	0.33043	+867.5
Surface U with a separated iron vacancy	-31897.38096	0.023504	-31897.40446	0.05533	+145.3
Near-surface U with a separated iron vacancy	-31897.44399	0.01581	-31897.45679	0.00000	0.0
Surface U with no iron vacancy	-33160.30268	0.02613	-33160.32881	0.00000	0.0

Near-surface U with no iron vacancy	-33160.07085	0.01679	-33160.08764	0.24117	+633.2

Table S4: Comparative energy calculations for solvated initial U(VI) incorporation into [010] goethite.

Model	Final oxidation of U	E in Hartree	Gibbs correction to E in Hartree	New E in Hartree	Relative E in Hartree	Relative E in kJ/mol
Surface U with an adjacent iron vacancy	U(VI)	-32965.63769	0.07702	-32965.71471	0.00000	0.0
Near-surface U with an adjacent iron vacancy	U(VI)	-32965.34655	0.07600	-32965.42255	0.29216	+767.1
Surface U with an adjacent iron vacancy	U(V)	-32965.61175	0.07763	-32965.68938	0.02533	+66.5
Near-surface U with an	U(V)	-32965.32922	0.07595	-32965.40517	0.30953	+812.7

adjacent iron vacancy						
Surface U with a separated iron vacancy	U(V)	-32965.55515	0.07718	-32965.63200	0.08271	+217.2
Near-surface U with a separated iron vacancy	U(V)	-32965.62506	0.07811	-32965.70300	0.01171	+30.7
Surface U with no iron vacancy	U(V)	-34228.08331	0.07651	-34228.15982	0.00000	0.0
Near-surface U with no iron vacancy	U(V)	-34227.86008	0.06557	-34227.92565	0.23418	+614.8

Table S5: Comparative energy calculations for solvated U(V) incorporation into [010] goethite.

Model	E in Hartree	Gibbs correction to E in Hartree	New E in Hartree	Relative E in Hartree	Relative E in kJ/mol
Surface U with an adjacent iron vacancy	-32965.83939	0.07433	-32965.91333	0.00000	0.0
Near-surface U with an adjacent iron vacancy	-32965.54085	0.07308	-32965.61393	0.29636	+787.1
Surface U with a separated iron vacancy	-32965.77967	0.07855	-32965.85822	0.05550	+145.7
Near-surface U with a separated iron vacancy	-32965.82327	0.07496	-32965.89823	0.01549	+40.7
Surface U with no iron vacancy	-34228.56786	0.08023	-34228.64809	0.00000	0.0

Near-surface U with no iron vacancy	-34228.29786	0.07108	-34228.36894	0.27915	+732.9

Table S6: Comparative energy calculations for solvated U(IV) incorporation into [010] goethite.

Model	E in Hartree	Gibbs correction to E in Hartree	New E in Hartree	Relative E in Hartree	Relative E in kJ/mol
Surface U with an adjacent iron vacancy	-32965.89519	0.07656	-32965.97175	0.00528	+13.8
Near-surface U with an adjacent iron vacancy	-32965.59629	0.06644	-32965.66274	0.31428	+825.2
Surface U with a separated iron vacancy	-32965.85050	0.07480	-32965.92531	0.05171	+135.8

Near-surface U with a separated iron vacancy	-32965.90177	0.07525	-32965.97702	0.00000	0.0
Surface U with no iron vacancy	-34228.89930	0.07553	-34228.97546	0.00000	0.0
Near-surface U with no iron vacancy	-34228.63946	0.07552	-34228.71501	0.26045	+683.8

Section 2: Complete set of unsolvated incorporation data

During this study a wide variety of uranium incorporation schemes were tested by altering the position of incorporated uranium species, iron vacancies and oxidation state. As such, there are too many data for the main body of the paper, and therefore only the key results were discussed. In this section, the best optimised geometries for each oxidation state, uranium position and iron vacancy combination are given, showing how all of these can and do affect how uranium incorporates into goethite. For all tables, the calculated relative energy is given, based on SI section 1. Spin density was used to determine the number of unpaired electrons, and thus oxidation state.

Table S7: U-O bonds lengths (Å) obtained following initial U(VI) incorporation into the surface and near-surface of [010] goethite, using the numbering given in Figures 2a) and b) within the main text. Spin density on the U also shown and used to determine the final oxidation state. Relative energies in kJ/mol.

U position	Vacancy position		U-O1	U-O2	U-O3	U-O4	U-O5	U-O6	Average U-O	Spin density of U	Final oxidation state	Relative energy
Surface	Adjacent	Edge - sharing	1.81	1.91	2.06	2.17	2.14		2.02	0.12	U(VI)	0.0
Near-surface	Adjacent	Corner-sharing	2.28	1.86	2.05	1.97	2.29	2.08	2.09	0.08	U(VI)	+821.0
Surface	Adjacent	Edge-sharing	2.00	1.94	2.14	2.19	2.18		2.09	-1.05	U(V)	+58.3
Near-surface	Adjacent	Corner-Sharing	2.41	1.90	2.08	2.45	2.46	2.03	2.22	-0.98	U(V)	+870.4
Surface	Separated		2.08	1.98	2.08	2.12	2.12		2.08	-1.06	U(V)	+226.3
Near-surface	Separated		2.12	2.09	2.04	2.21	2.23	2.11	2.13	-1.06	U(V)	+79.8
Surface	No vacancy		1.97	2.08	2.04	2.10	2.19		2.08	-1.05	U(V)	0.0
Near-surface	No vacancy		2.09	2.06	2.06	2.08	2.31	2.11	2.12	-1.05	U(V)	+467.3

Table S8: U-O bonds lengths (Å) obtained following initial U(V) incorporation into the surface and near-surface of [010] goethite, using the numbering given in Figures 2a) and b) within the main text. Spin density located on the U also shown and used to determine the final oxidation state. Relative energies in kJ/mol.

U position	Vacancy position	U-O1	U-O2	U-O3	U-O4	U-O5	U-O6	Average U-O	Spin density of U	Final valency	Relative energy
Surface	Adjacent	1.99	1.95	2.16	2.21	2.15		2.09	1.25	U(V)	+28.3
Near-surface	Adjacent	2.27	1.92	2.09	2.27	2.31	2.08	2.16	1.17	U(V)	+830.5
Surface	Separated	2.09	1.92	2.12	2.15	2.12		2.08	1.25	U(V)	+166.4
Near-surface	Separated	2.12	2.06	2.06	2.23	2.24	2.11	2.14	1.22	U(V)	0.0
Surface	No vacancy	1.97	2.08	2.04	2.10	2.19		2.08	1.23	U(V)	0.0
Near-surface	No vacancy	2.23	2.03	2.05	2.06	2.31	2.08	2.13	1.22	U(V)	+571.2

Table S9: U-O bonds lengths (Å) obtained following initial U(IV) incorporation into the surface and near-surface of [010] goethite, using the numbering given in Figures 2a) and b) within the main text. Spin density on the U also shown and used to determine the final oxidation state. Relative energies in kJ/mol.

U position	Vacancy position	U-O1	U-O2	U-O3	U-O4	U-O5	U-O6	Average U-O	Spin density of U	Final valency	Relative energy
Surface	Adjacent	2.08	2.03	2.35	2.33	2.26		2.21	2.18	U(IV)	+66.6
Near-surface	Adjacent	2.39	2.01	2.18	2.42	2.41	2.16	2.26	2.11	U(IV)	+867.5
Surface	Separated	2.22	2.01	2.23	2.27	2.19		2.18	2.19	U(IV)	+145.3
Near-surface	Separated	2.22	2.18	2.16	2.33	2.31	2.19	2.23	2.13	U(IV)	0.0
Surface	No vacancy	2.23	2.15	2.12	2.17	2.22		2.18	2.16	U(IV)	0.0
Near-surface	No vacancy	2.31	2.12	2.11	2.31	2.44	2.12	2.23	2.13	U(IV)	+633.2

Section 3: Solvation data

Following solvation of the [010] goethite surface, a new set of optimised geometries for each model was calculated. For surface uranium incorporation an additional U-O bond length was found due to the presence of the explicit water. For comparison to unsolvated data, this U-OH_2 bond length is not included in the average U-O bond length. For all tables, the calculated relative energy is given, based on SI section 1

Table S10: U-O bonds lengths (Å) calculated for U(VI) incorporation into the surface and near-surface of solvated [010] goethite, using the numbering given in Figures 2a) and b) within the main text. Where U-O7 represents the connections between surface U and water. Final predicted valency determined natural spin density analysis. Relative energies in kJ/mol.

U position	Vacancy position	U-O1	U-O2	U-O3	U-O4	U-O5	U-O6	U-O7	U-O average	Spin density of U	Final valency	Relative energy
Surface	Adjacent vacancy	1.84	1.93	2.05	2.18	2.14		2.51	2.03	0.12	U(VI)	0.0
Near-surface	Adjacent vacancy	2.22	1.87	2.04	1.98	2.24	2.02		2.06	0.08	U(VI)	+767.1
Surface	Adjacent vacancy	2.01	1.95	2.14	2.22	2.18		2.51	2.10	-1.03	U(V)	+66.5
Near-surface	Adjacent vacancy	2.31	2.00	2.17	2.31	2.34	2.34		2.25	-0.97	U(V)	+812.7
Surface	Separated Vacancy	1.96	2.03	2.08	2.19	2.18		2.53	2.09	-1.07	U(V)	+217.2
Near-surface	Separated Vacancy	2.12	2.09	2.07	2.24	2.23	2.04		2.13	-1.05	U(V)	+30.7
Surface	No vacancy	2.05	2.09	2.08	1.99	2.24		2.51	2.08	-1.23	U(V)	0.0
Near-surface	No vacancy	2.10	2.05	2.06	2.10	2.30	2.08		2.12	-1.05	U(V)	+614.8

Table S11: U-O bonds lengths (Å) calculated for U(V) incorporation into the surface and near-surface of solvated [010] goethite, using the numbering given in Figures 2a) and b) within the main text. Where U-O7 represents the connections between surface U and water. Final predicted valency determined natural spin density analysis. Relative energies in kJ/mol.

U position	Vacancy position	U-O1	U-O2	U-O3	U-O4	U-O5	U-O6	U-O7	U-O average	Spin density of U	Final valency	Relative energy
Surface	Adjacent vacancy	2.01	1.98	2.15	2.23	2.13		2.52	2.10	1.25	U(V)	0.0
Near-surface	Adjacent vacancy	2.25	1.94	2.10	2.25	2.28	2.02		2.14	1.20	U(V)	+787.1
Surface	Separated Vacancy	1.89	2.04	2.11	2.24	2.21		2.54	2.10	1.22	U(V)	+145.7
Near-surface	Separated Vacancy	2.12	2.06	2.09	2.26	2.23	2.04		2.13	1.22	U(V)	+40.7
Surface	No vacancy	2.05	2.06	2.07	2.00	2.25		2.51	2.09	1.23	U(V)	0.0
Near-surface	No vacancy	2.22	2.02	2.02	2.22	2.32	2.01		2.14	1.22	U(V)	+732.9

Table S12: U-O bonds lengths (Å) calculated for U(IV) incorporation into the surface and near-surface of solvated [010] goethite, using the numbering given in Figures 2a) and b) within the main text. Where U-O7 represents the connections between surface U and water. Final predicted valency determined natural spin density analysis. Relative energies in kJ/mol.

U position	Vacancy position	U-O1	U-O2	U-O3	U-O4	U-O5	U-O6	U-O7	U-O average	Spin density of U	Final valency	Relative energy
Surface	Adjacent vacancy	2.17	2.04	2.28	2.35	2.20		2.61	2.21	2.16	U(IV)	+13.8
Near-surface	Adjacent vacancy	2.41	2.01	2.17	2.42	2.41	2.08		2.25	2.13	U(IV)	+825.2
Surface	Separated Vacancy	2.11	2.07	2.21	2.31	2.26		2.61	2.19	2.15	U(IV)	+135.8
Near-surface	Separated Vacancy	2.25	2.18	2.16	2.37	2.34	2.10		2.23	2.13	U(IV)	0.0
Surface	No vacancy	2.23	2.12	2.13	2.20	2.27		2.57	2.19	2.15	U(IV)	0.0
Near-surface	No vacancy	2.32	2.10	2.11	2.33	2.40	2.07		2.22	2.14	U(IV)	+683.8

Section 4: Calculated frequency data

For each model and oxidation state, frequencies were calculated. Here we provide these data for the following models (both unsolvated and solvated).

1. U(VI) incorporation into the surface of [010] goethite with an adjacent vacancy
2. U(VI) reduction and U(V) incorporation into the surface of [010] goethite with an adjacent vacancy
3. U(VI) reduction and U(V) incorporation into the near-surface of [010] goethite with a separated vacancy
4. U(V) incorporation into the surface of [010] goethite with an adjacent vacancy
5. U(V) incorporation into the near-surface of [010] goethite with a separated vacancy
6. U(IV) incorporation into the surface of [010] goethite with an adjacent vacancy
7. U(IV) incorporation into the near-surface of [010] goethite with a separated vacancy

For models used in this research but not listed above, the frequency data have been calculated and are available upon request.

Table S13: Calculated frequencies for U(VI) incorporation into the surface of [010] goethite with an adjacent iron vacancy. System belongs to the C_1 point group.

Mode	Wavenumber/ cm^{-1}	IR intensity/ $kmmol^{-1}$
1	91.74	2.71789
2	101.11	0.84431
3	115.61	2.24162
4	152.92	23.03959
5	170.86	8.39938
6	188.92	25.87909
7	208.69	10.73621
8	230.91	24.98727
9	234.57	33.21344
10	241.22	9.48248
11	269.14	3.30419
12	282.54	10.59472
13	296.63	18.53242
14	297.37	19.27376
15	315.00	51.07554
16	329.50	11.73253
17	336.88	31.16622
18	341.79	21.72780
19	353.18	29.48787
20	385.12	22.26135
21	395.12	37.16810

22	409.04	51.26895
23	432.26	176.90381
24	444.59	75.12771
25	451.62	6.11283
26	452.19	9.52876
27	457.25	7.64350
28	480.07	117.75032
29	488.16	100.39925
30	508.59	42.54601
31	536.69	47.45427
32	547.06	89.73511
33	549.02	25.14386
34	601.42	122.21295
35	635.49	48.02409
36	658.13	81.89825
37	749.68	425.42127
38	809.08	283.20550
39	861.56	99.58807
40	939.11	531.26824
41	947.72	168.61137
42	1019.97	56.14598
43	1087.23	251.86249
44	1117.50	279.28695
45	1154.58	398.01149

46	1289.32	104.48066
47	1407.44	234.35452
48	2268.65	3373.12699
49	3144.38	1453.17634
50	3224.51	742.24464
51	3594.25	1483.38315

Table S14: Calculated frequencies for U(VI) and reduction leading to U(V) incorporation into the surface of [010] goethite with an adjacent iron vacancy. System belongs to the C_1 point group.

Mode	Wavenumber/ cm^{-1}	IR intensity/ $kmmol^{-1}$
1	100.76	1.29351
2	105.05	0.15106
3	113.17	0.59826
4	166.67	0.84608
5	192.46	1.72572
6	218.51	0.77936
7	236.33	7.95868
8	243.23	6.60170
9	258.02	6.00001
10	261.63	2.14906
11	272.49	5.70898
12	300.35	0.88188
13	317.89	14.43116
14	333.07	13.32069
15	335.97	16.15457
16	350.65	12.17019
17	377.10	29.60576
18	382.67	43.98714
19	394.26	25.42251
20	407.22	39.08136

21	410.66	2.16738
22	419.17	15.36984
23	459.92	244.17357
24	471.73	22.00449
25	486.47	59.56837
26	510.86	29.37691
27	514.50	16.21440
28	520.95	32.16035
29	528.75	94.22252
30	538.71	316.70844
31	561.72	68.15842
32	582.15	186.08771
33	584.90	116.20211
34	603.11	31.54650
35	614.56	212.67844
36	620.94	148.20455
37	682.35	178.28432
38	788.66	201.88518
39	984.53	31.15238
40	993.90	147.96299
41	1033.73	72.95348
42	1096.68	130.28189
43	1146.54	38.28485
44	1188.47	50.06131

45	1220.46	98.08566
46	1290.62	84.08506
47	1387.23	194.63852
48	2885.88	1097.22126
49	3156.14	870.35393
50	3218.03	1189.23159
51	3341.58	944.87846

Table S15: Calculated frequencies for U(VI) and reduction for U(V) incorporation into the near-surface of [010] goethite with a separated iron vacancy. System belongs to the C_1 point group.

Mode	Wavenumber/ cm^{-1}	IR intensity/ $kmmol^{-1}$
1	70.16	1.43560
2	106.18	2.02329
3	119.33	2.24815
4	126.68	12.39094
5	126.88	0.30066
6	144.67	31.15709
7	165.57	4.27196
8	169.14	7.75582
9	174.33	6.27701
10	196.46	27.75346
11	206.94	18.87872
12	219.22	6.73002
13	221.31	1.33065
14	241.22	5.14751
15	258.62	4.56098
16	276.38	28.96546
17	298.91	5.22927
18	307.00	11.08453
19	309.87	24.87516
20	330.08	37.71116

21	352.57	31.07493
22	392.86	20.77611
23	397.11	33.16217
24	432.31	9.70416
25	435.40	99.59263
26	455.83	48.41451
27	490.05	57.74588
28	491.78	71.96560
29	501.09	18.29949
30	514.66	135.99402
31	520.44	42.41953
32	536.19	248.67038
33	550.38	72.62738
34	561.05	208.48999
35	585.16	262.40646
36	615.02	170.13845
37	634.33	80.13972
38	695.32	13.55746
39	702.33	99.28182
40	970.40	32.78434
41	1018.05	84.07244
42	1071.10	62.73780
43	1084.86	45.26250
44	1100.37	472.14871

45	1122.60	198.62014
46	1159.81	5.78293
47	1404.58	168.26525
48	2433.55	274.80882
49	2499.69	2162.77952
50	3188.60	1655.26481
51	3699.02	617.88756

Table S16: Calculated frequencies for U(V) incorporation into the surface of [010] goethite with an adjacent iron vacancy. System belongs to the C_1 point group.

Mode	Wavenumber/ cm^{-1}	IR intensity/ $kmmol^{-1}$
1	92.72	8.84101
2	96.09	34.82539
3	108.91	60.93988
4	124.36	120.58278
5	161.84	38.14758
6	172.35	388.20951
7	190.30	16.61764
8	200.23	24.11554
9	213.46	80.68467
10	242.02	127.78866
11	248.44	80.92671
12	274.94	48.78851
13	278.51	3.89112
14	287.77	253.57852
15	292.38	307.67178
16	325.32	20.45167
17	326.02	19.88961
18	334.57	36.37512
19	349.27	55.72966
20	366.07	4.37481
21	368.76	162.00980

22	402.03	228.69974
23	426.15	253.64002
24	434.33	147.29561
25	438.47	41.41645
26	444.82	85.39135
27	453.42	42.55298
28	478.64	293.13196
29	483.83	10.05926
30	490.90	26.42362
31	506.97	479.49480
32	542.09	63.64220
33	545.03	78.45003
34	572.73	315.82064
35	592.16	248.27115
36	652.36	479.75843
37	669.89	149.82115
38	761.44	367.25055
39	772.37	542.84625
40	886.16	1094.96717
41	915.21	1737.09573
42	940.57	136.58219
43	998.78	741.21286
44	1059.28	327.42764
45	1118.86	21.76304

46	1274.48	901.08538
47	1408.08	161.19908
48	2145.82	2672.92483
49	2869.88	1472.24205
50	3323.09	1663.41500
51	3535.00	2815.07397

Table S17: Calculated frequencies for U(V) incorporation into the near-surface of [010] goethite with a separated iron vacancy. System belongs to the C_1 point group.

Mode	Wavenumber/ cm^{-1}	IR intensity/ $kmmol^{-1}$
1	80.90	1.50323
2	117.38	0.12113
3	129.22	0.26013
4	156.05	1.48695
5	171.26	2.18163
6	185.82	7.17675
7	193.84	6.93560
8	213.55	17.73894
9	227.23	16.51209
10	234.40	14.26730
11	256.62	0.20630
12	266.02	4.45815
13	271.37	5.03581
14	295.71	10.90895
15	318.73	4.15861
16	329.46	7.32257
17	340.11	37.64337
18	363.49	11.50438
19	365.35	13.99694
20	373.14	22.98668
21	394.26	46.33305

22	403.55	44.68466
23	428.87	19.03452
24	474.87	100.34970
25	484.12	36.88133
26	487.95	21.62437
27	505.94	18.10977
28	520.09	102.60509
29	522.99	22.84865
30	533.19	77.79694
31	575.09	155.24421
32	594.76	312.78991
33	602.58	114.72199
34	608.52	335.80004
35	609.39	12.77591
36	677.17	93.77167
37	712.68	33.38626
38	819.46	136.90792
39	920.48	55.13377
40	951.97	192.72516
41	1047.83	85.85328
42	1136.02	81.54555
43	1212.22	92.80056
44	1250.08	174.66020
45	1256.04	92.26695

46	1328.08	31.06343
47	1366.79	202.77890
48	2498.51	946.33261
49	2638.52	1522.75352
50	3251.27	1764.89631
51	3736.37	597.53238

Table S18: Calculated frequencies for U(IV) incorporation into the surface of [010] goethite with an adjacent iron vacancy. System belongs to the C_1 point group.

Mode	Wavenumber/ cm^{-1}	IR intensity/ $kmmol^{-1}$
1	70.22	4.75450
2	80.92	3.26128
3	93.71	12.43432
4	98.62	1.71195
5	99.07	3.53306
6	120.39	4.60297
7	167.29	0.21903
8	181.97	2.81290
9	201.66	12.73568
10	217.48	3.43943
11	232.14	24.21429
12	255.01	5.32246
13	269.46	21.60750
14	274.43	4.06661
15	280.77	31.21254
16	292.59	11.04099
17	293.33	0.19815
18	327.64	41.71847
19	338.29	26.76929
20	357.26	7.09769
21	374.58	25.75388

22	391.26	138.50361
23	402.93	59.92139
24	435.93	97.64467
25	443.80	122.53081
26	459.03	66.55371
27	464.93	32.97138
28	471.32	181.55781
29	491.65	34.54597
30	495.03	89.25115
31	531.52	114.93403
32	541.94	72.23655
33	558.80	190.23429
34	565.63	2.42712
35	573.34	197.60366
36	602.59	94.90629
37	613.68	161.15319
38	640.59	61.68645
39	721.06	190.00314
40	727.13	106.57863
41	759.99	94.24294
42	817.85	175.93836
43	971.79	67.59471
44	979.78	30.33701
45	1039.26	62.92133

46	1227.01	174.45372
47	1354.93	187.83228
48	2532.80	2051.97178
49	3463.78	696.92290
50	3620.57	277.60323
51	3822.84	210.03005

Table S19: Calculated frequencies for U(IV) incorporation into the near-surface of [010] goethite with a separated iron vacancy. System belongs to the C_1 point group.

Mode	Wavenumber/ cm^{-1}	IR intensity/ $kmmol^{-1}$
1	60.92	2.51026
2	99.33	12.04532
3	116.44	2.32174
4	118.81	1.67359
5	122.56	12.04031
6	137.30	8.11179
7	155.33	13.30778
8	171.87	27.49246
9	195.24	23.13978
10	202.41	12.53096
11	221.73	2.83180
12	231.55	2.51875
13	241.29	4.50550
14	260.12	3.11608
15	280.46	13.96474
16	306.75	0.77372
17	317.04	15.93685
18	330.70	0.99587
19	340.33	25.10796
20	356.11	39.46987
21	370.57	26.17388

22	381.59	23.32911
23	392.49	32.34384
24	434.73	13.30170
25	445.79	159.55838
26	456.57	33.89933
27	461.05	47.75552
28	469.35	65.92153
29	478.50	36.72328
30	492.34	155.54850
31	511.13	28.26741
32	514.51	99.75935
33	527.11	131.63465
34	560.55	340.22517
35	569.07	121.80253
36	632.73	92.92129
37	635.50	17.38850
38	691.12	175.99375
39	830.81	51.45847
40	838.33	294.25101
41	962.28	105.50487
42	1016.93	267.36165
43	1034.49	234.99225
44	1050.14	67.18790
45	1118.40	158.74580

46	1150.18	52.10762
47	1377.90	189.03044
48	3055.08	1815.06859
49	3059.56	1452.14552
50	3167.75	1106.72134
51	3665.59	498.35962

Table S20: Calculated frequencies for solvated U(VI) incorporation into the surface of [010] goethite with an adjacent iron vacancy. System belongs to the C_1 point group.

Mode	Wavenumber/ cm^{-1}	IR intensity/ $kmmol^{-1}$
1	87.34	0.29364
2	100.49	0.17668
3	112.50	2.33270
4	122.46	0.30618
5	124.71	1.70558
6	141.40	1.27669
7	147.94	4.70522
8	159.18	14.24905
9	162.17	1.02962
10	173.71	8.82687
11	228.38	1.51352
12	261.18	10.87401
13	269.09	18.76018
14	303.35	14.65234
15	312.91	4.62845
16	313.84	13.97690
17	314.45	22.77226
18	318.44	2.10722
19	340.05	11.65274
20	341.97	14.75012
21	346.69	10.30539

22	348.89	13.73157
23	351.67	20.93481
24	370.53	35.10381
25	392.22	20.23585
26	407.72	44.20664
27	417.31	18.67003
28	449.20	9.86361
29	462.30	128.17710
30	475.00	133.24269
31	480.92	45.48345
32	511.61	50.99089
33	515.83	22.76451
34	527.22	27.39597
35	531.45	13.29794
36	543.84	18.57812
37	544.47	17.62659
38	572.73	229.86699
39	588.21	52.23122
40	609.19	20.11344
41	641.27	238.20581
42	662.51	149.53568
43	691.53	77.68743
44	695.68	139.58777
45	739.47	196.48615

46	769.37	519.31612
47	777.95	78.44331
48	858.91	5.89244
49	860.59	163.42824
50	870.47	131.06082
51	1023.52	128.55607
52	1025.93	70.65572
53	1085.22	42.19982
54	1169.02	210.18685
55	1174.50	41.02268
56	1192.39	120.40183
57	1275.12	44.02814
58	1323.27	21.02534
59	1429.66	158.21716
60	1664.45	56.42282
61	1704.55	67.89868
62	2508.23	1405.48370
63	3330.13	700.48065
64	3390.23	1461.54851
65	3431.64	907.05961
66	3675.93	270.61051
67	3809.71	88.10728
68	3881.78	156.17263
69	3953.03	113.29749

Table S21: Calculated frequencies for solvated U(VI) and reduction leading to U(V) incorporation into the surface of [010] goethite with an adjacent iron vacancy. System belongs to the C_1 point group.

Mode	Wavenumber/ cm^{-1}	IR intensity/ $kmmol^{-1}$
1	85.31	0.28609
2	102.37	1.24007
3	115.28	1.10823
4	123.47	0.92251
5	123.90	0.26248
6	140.03	2.73651
7	162.03	5.84958
8	166.74	4.25374
9	168.03	1.67409
10	176.34	11.82431
11	234.17	0.46342
12	267.24	7.53733
13	278.27	18.83208
14	290.60	6.60474
15	306.19	11.57942
16	308.93	11.65746
17	317.47	5.36065
18	319.18	18.08398
19	328.54	5.70026
20	342.15	21.71500

21	356.47	12.10694
22	356.94	13.01974
23	365.35	52.43660
24	391.14	30.96748
25	404.39	40.76996
26	410.65	36.23590
27	428.20	75.35366
28	440.92	19.24465
29	467.52	104.99135
30	495.32	186.68295
31	511.55	11.53743
32	511.97	51.80492
33	514.78	28.93258
34	528.02	39.87187
35	542.26	229.41238
36	548.12	14.43633
37	577.11	52.63399
38	602.13	277.96109
39	607.72	82.30551
40	627.69	132.14740
41	659.01	152.80718
42	691.41	100.92765
43	703.71	173.42237
44	714.84	163.47961

45	745.13	119.16952
46	758.76	269.55155
47	790.91	26.93911
48	797.58	235.42407
49	846.00	48.39367
50	886.01	113.81176
51	1033.22	142.82440
52	1035.35	52.53123
53	1151.69	89.87377
54	1185.15	90.00853
55	1193.25	45.44579
56	1265.26	40.50453
57	1281.17	118.61691
58	1292.84	42.53895
59	1440.78	103.74698
60	1673.27	74.68324
61	1712.98	72.77651
62	2741.61	318.60994
63	2800.55	2201.74791
64	3324.89	1068.69301
65	3413.26	1324.44270
66	3729.14	186.06882
67	3827.83	84.06755
68	3858.97	256.97240

69	3962.31	129.06247

Table S22: Calculated frequencies for solvated U(VI) and reduction for U(V) incorporation into the near-surface of [010] goethite with a separated iron vacancy. System belongs to the C_1 point group.

Mode	Wavenumber/ cm^{-1}	IR intensity/ $kmmol^{-1}$
1	98.44	1.85462
2	124.30	0.40043
3	147.54	0.09161
4	163.93	10.39678
5	171.30	5.51403
6	178.57	1.99074
7	193.88	1.39497
8	202.41	1.84787
9	203.51	2.22785
10	219.93	3.05124
11	224.66	5.55664
12	231.22	4.11069
13	234.42	12.88471
14	241.70	21.17290
15	267.16	14.20131
16	279.61	7.55352
17	284.85	6.25137
18	298.52	19.61245
19	307.11	38.24048
20	320.19	13.08222

21	335.77	13.28062
22	340.86	5.64826
23	351.94	25.92500
24	364.40	18.69097
25	376.61	21.02253
26	398.57	40.59855
27	405.90	11.12511
28	419.64	43.10735
29	427.60	25.08213
30	476.50	136.49715
31	499.03	18.45424
32	499.62	37.67940
33	509.81	33.98418
34	521.83	9.76381
35	532.05	117.06448
36	546.71	40.83388
37	568.62	83.39046
38	571.19	269.09181
39	605.01	224.51931
40	633.06	129.20557
41	645.88	199.94895
42	659.07	101.04749
43	706.34	52.31829
44	749.76	56.11256

45	782.89	152.62370
46	820.53	234.00749
47	834.19	142.06512
48	862.66	87.10020
49	881.28	139.12037
50	1061.02	78.76827
51	1117.48	60.70610
52	1153.33	55.03297
53	1171.75	105.12141
54	1230.63	110.94881
55	1294.42	38.37162
56	1303.28	125.27247
57	1314.41	218.50880
58	1383.40	18.07068
59	1399.77	217.73848
60	1687.52	192.15246
61	1697.85	1.65118
62	2387.25	1059.37438
63	2572.47	1580.79093
64	3157.48	1150.02686
65	3460.45	998.01027
66	3755.05	150.61704
67	3773.73	104.73701
68	3826.65	185.90331

69	3850.87	247.77855

Table S23: Calculated frequencies for solvated U(V) incorporation into the surface of [010] goethite with an adjacent iron vacancy. System belongs to the C_1 point group.

Mode	Wavenumber/ cm^{-1}	IR intensity/ $kmmol^{-1}$
1	103.29	0.82968
2	110.95	1.58226
3	112.14	27.53413
4	118.84	0.78087
5	132.02	20.21090
6	153.45	17.37793
7	163.05	5.64924
8	166.70	1.08545
9	175.75	4.52460
10	233.18	7.99708
11	243.14	2.54693
12	269.50	25.13168
13	269.71	4.25303
14	276.20	17.21844
15	283.63	8.24658
16	304.03	38.70472
17	307.82	10.96789
18	311.23	12.79627
19	318.74	5.47934
20	328.05	16.11732
21	342.37	5.75841

22	349.50	26.29550
23	362.08	17.73875
24	367.00	46.62074
25	375.57	58.55846
26	399.13	66.65214
27	408.10	66.21924
28	421.48	43.91729
29	452.03	240.67580
30	460.60	81.22583
31	486.79	37.74202
32	496.40	46.06535
33	501.63	17.14959
34	510.17	15.36690
35	516.04	33.20810
36	540.72	14.15981
37	567.61	59.81507
38	584.85	112.06807
39	590.06	210.35140
40	601.12	185.30957
41	606.30	238.65411
42	628.72	107.15441
43	640.82	253.19921
44	679.14	72.01948
45	698.88	290.37396

46	750.33	200.55997
47	766.84	120.74967
48	815.49	233.44689
49	873.87	69.19448
50	993.36	122.97025
51	1039.81	77.84916
52	1095.81	429.66319
53	1172.25	76.70668
54	1206.99	87.78620
55	1220.97	171.74736
56	1232.54	345.27653
57	1247.60	110.36252
58	1330.24	206.17132
59	1433.56	229.36601
60	1665.35	18.88657
61	1714.38	254.92599
62	2220.01	874.89916
63	2780.66	1079.92298
64	3057.91	1847.43609
65	3337.84	1583.30821
66	3593.14	107.27248
67	3712.91	256.90858
68	3866.12	238.78534
69	3886.68	208.86735

Table S24: Calculated frequencies for solvated U(V) incorporation into the near-surface of [010] goethite with a separated iron vacancy. System belongs to the C_1 point group.

Mode	Wavenumber/ cm^{-1}	IR intensity/ $kmmol^{-1}$
1	89.70	2.60436
2	115.97	0.27075
3	139.83	0.29021
4	157.29	13.75764
5	166.10	2.87423
6	170.52	2.42460
7	189.18	0.76865
8	193.42	2.67757
9	200.78	3.64867
10	201.80	4.40484
11	211.33	20.58807
12	216.90	3.98208
13	226.02	15.94641
14	231.69	10.23235
15	255.26	18.52991
16	263.25	2.12363
17	270.15	10.61047
18	290.75	23.51284
19	307.72	12.53551
20	309.93	5.94372
21	320.15	2.71725

22	331.84	10.91847
23	341.43	40.21525
24	354.51	16.87621
25	371.77	15.44553
26	382.03	37.15646
27	391.34	18.77558
28	402.21	44.11300
29	407.03	9.83799
30	439.71	115.23501
31	481.67	4.25409
32	483.59	80.21300
33	491.89	34.82185
34	505.12	7.57500
35	518.95	103.41502
36	528.15	82.44723
37	564.06	218.62905
38	589.56	252.29770
39	606.13	84.11918
40	616.01	260.45281
41	639.07	34.71379
42	670.99	47.80367
43	678.61	71.30636
44	738.15	42.16063
45	754.82	161.59210

46	780.43	264.40664
47	796.91	156.21766
48	829.28	59.10548
49	859.67	116.25082
50	1019.80	189.94772
51	1063.68	76.85743
52	1105.48	87.27343
53	1151.25	56.39570
54	1194.67	63.63002
55	1251.28	96.81296
56	1268.37	99.72878
57	1291.79	156.62501
58	1344.33	28.68728
59	1358.93	189.28859
60	1675.55	187.95614
61	1690.78	1.64099
62	2417.07	1211.73421
63	2685.65	1252.56296
64	3103.03	1902.77046
65	3330.24	1257.44404
66	3754.62	150.88037
67	3778.58	79.87282
68	3829.68	191.03165
69	3859.93	222.14419

Table S25: Calculated frequencies for solvated U(IV) incorporation into the surface of [010] goethite with an adjacent iron vacancy. System belongs to the C_1 point group.

Mode	Wavenumber/ cm^{-1}	IR intensity/ $kmmol^{-1}$
1	93.52	1.90184
2	111.36	0.69879
3	116.37	0.28001
4	125.70	0.14515
5	132.63	0.90297
6	153.43	1.32692
7	160.00	12.45967
8	169.94	7.21298
9	173.28	5.02408
10	214.08	10.65775
11	226.15	3.38405
12	231.59	0.22484
13	233.63	13.48784
14	258.18	8.04668
15	272.52	15.58484
16	277.69	20.59791
17	303.97	18.46758
18	305.44	12.56394
19	318.48	11.00277
20	320.11	26.54642
21	336.44	24.98872

22	345.93	57.22269
23	351.42	12.10029
24	361.87	18.42464
25	378.03	61.04662
26	393.28	24.29640
27	400.15	30.25310
28	436.56	138.26263
29	442.51	19.46367
30	450.56	30.32544
31	462.11	155.07326
32	498.04	48.80276
33	507.52	247.95583
34	510.97	38.32920
35	514.70	27.04193
36	552.05	14.53241
37	555.96	150.91956
38	580.60	61.05578
39	603.22	94.82935
40	606.42	62.81825
41	617.45	365.22894
42	645.15	59.83121
43	662.96	93.23352
44	726.56	69.71515
45	747.89	133.19200

46	758.75	157.79366
47	788.71	102.32894
48	801.89	226.11848
49	819.13	253.47960
50	864.41	28.28876
51	912.81	110.10715
52	1029.53	63.41255
53	1055.73	135.51456
54	1164.47	81.27150
55	1204.13	38.74173
56	1227.78	61.96552
57	1236.26	65.75394
58	1271.96	83.83081
59	1445.23	98.41071
60	1704.35	145.83520
61	1706.02	44.24609
62	3125.94	526.14100
63	3142.54	1916.39834
64	3379.18	1031.07220
65	3516.98	631.67986
66	3610.66	433.26731
67	3758.42	185.65282
68	3848.77	210.08736
69	3952.71	95.44480

Table S26: Calculated frequencies for solvated U(IV) incorporation into the near-surface of [010] goethite with a separated iron vacancy. System belongs to the C_1 point group.

Mode	Wavenumber/ cm^{-1}	IR intensity/ $kmmol^{-1}$
1	79.03	1.01496
2	105.62	0.24064
3	131.99	13.79830
4	160.74	1.89770
5	163.81	1.44548
6	173.63	3.02848
7	185.97	4.17907
8	188.94	0.52271
9	190.78	10.43105
10	193.20	4.10025
11	201.25	74.27862
12	204.80	5.07785
13	219.33	25.04800
14	226.41	12.84048
15	231.50	15.36346
16	256.84	22.75385
17	262.14	5.71477
18	285.10	30.04099
19	304.80	4.99437
20	307.84	8.42945
21	327.70	24.30964

22	330.13	19.70616
23	339.53	8.88866
24	376.30	22.70636
25	381.46	41.54442
26	394.77	33.46691
27	400.23	54.89727
28	403.93	92.19645
29	406.61	10.54541
30	447.03	18.91765
31	474.98	87.66697
32	481.15	47.14753
33	482.00	104.41110
34	494.97	94.88924
35	500.14	70.35948
36	516.38	68.96170
37	520.74	36.11491
38	544.82	188.13791
39	583.32	153.80586
40	599.15	129.57587
41	619.67	35.99486
42	650.34	63.28606
43	681.59	47.69862
44	711.79	92.10481
45	751.09	163.64672

46	769.14	140.83428
47	801.62	306.82010
48	813.92	47.75409
49	840.16	89.63104
50	1033.82	203.30009
51	1057.15	196.28326
52	1085.71	64.76669
53	1137.64	39.72706
54	1151.45	132.13778
55	1194.85	153.87133
56	1211.77	51.62968
57	1260.90	92.41280
58	1278.96	54.26325
59	1376.23	315.93022
60	1673.32	150.66746
61	1686.26	1.70337
62	3005.83	1883.68747
63	3033.96	132.82541
64	3060.83	3762.14856
65	3393.62	869.30368
66	3749.70	104.97079
67	3788.33	62.35863
68	3835.75	144.94773
69	3866.67	222.66396

<u>Section 5: Bar charts comparing effect of solvation on calculated U-O bond lengths.</u>

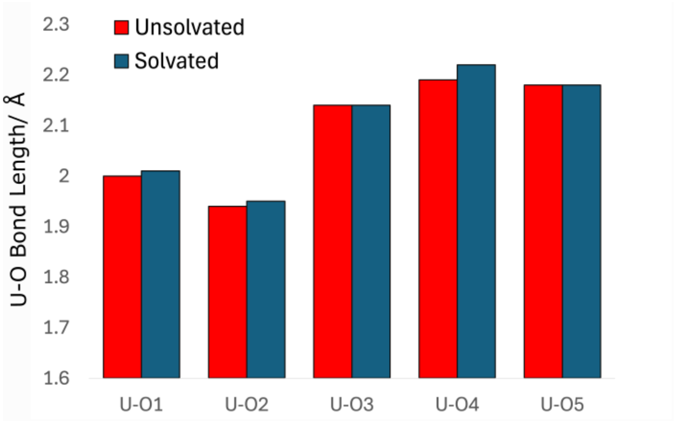

Figure S1: Bar chart comparing U-O bond lengths pre- and post-solvation for U(VI) incorporation and subsequent reduction to U(V) at the surface of [010] goethite with an adjacent iron vacancy.

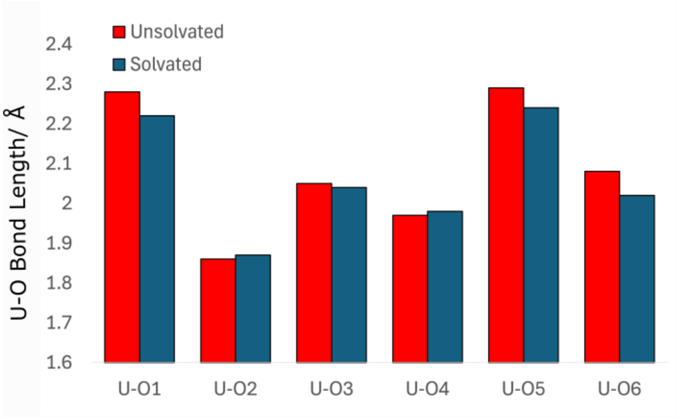

Figure S2: Bar chart comparing U-O bond lengths pre- and post-solvation for U(VI) incorporation at the near-surface of [010] goethite with an adjacent iron vacancy.

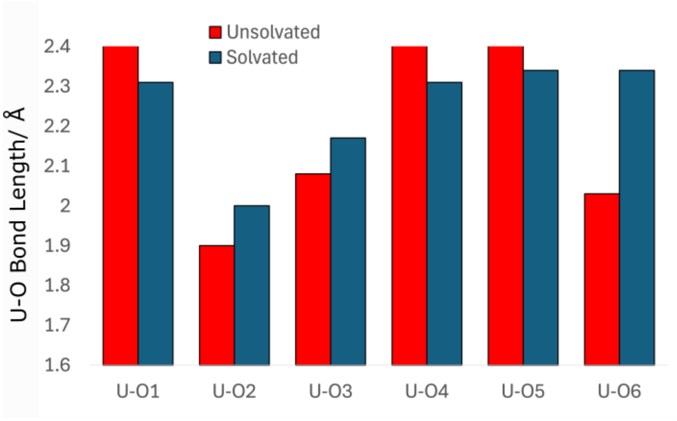

Figure S3: Bar chart comparing U-O bond lengths pre- and post-solvation for U(VI) incorporation and subsequent reduction to U(V) at the near-surface of [010] goethite with an adjacent iron vacancy.

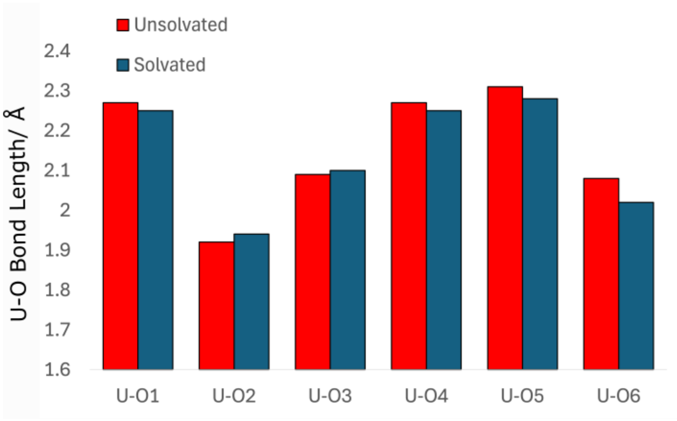

Figure S4: Bar chart comparing U-O bond lengths pre- and post-solvation for U(V) at the near-surface of [010] goethite with an adjacent iron vacancy.

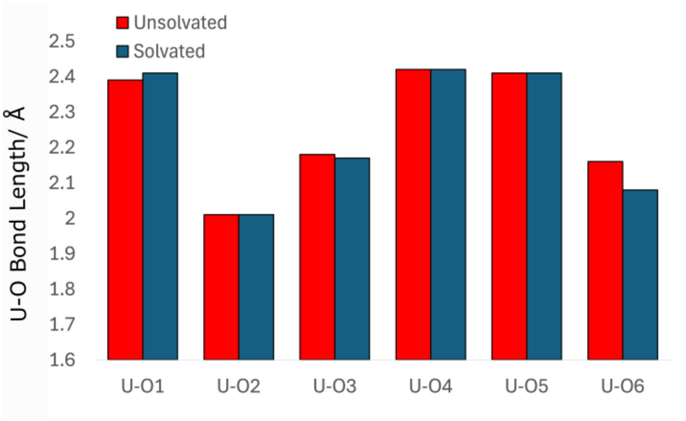

Figure S5: Bar chart comparing U-O bond lengths pre- and post-solvation for U(IV) incorporation at the near-surface of [010] goethite with an adjacent iron vacancy.

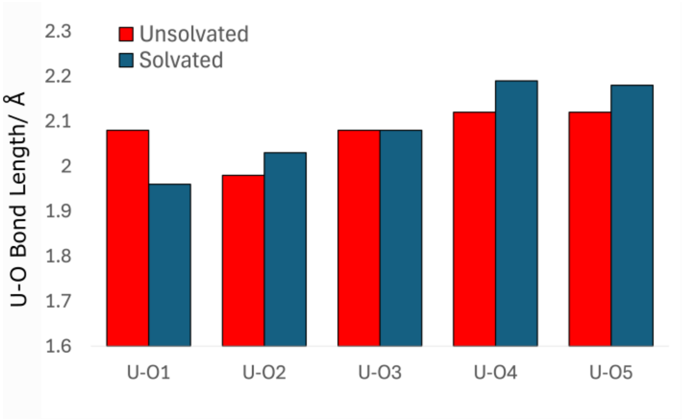

Figure S6: Bar chart comparing U-O bond lengths pre- and post-solvation for U(VI) incorporation and subsequent reduction to U(V) at the surface of [010] goethite with a separated iron vacancy.

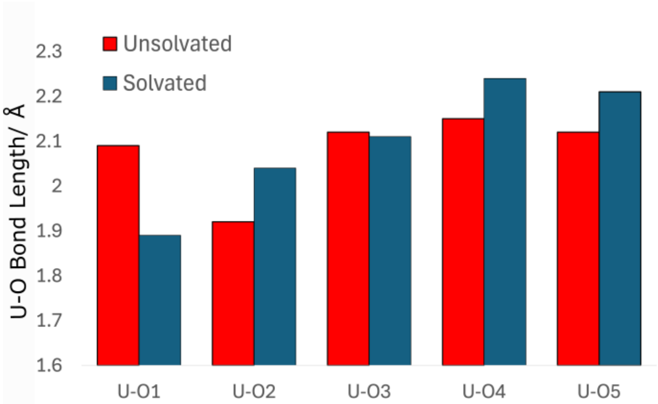

Figure S7: Bar chart comparing U-O bond lengths pre- and post-solvation for U(V) at the surface of [010] goethite with a separated iron vacancy.

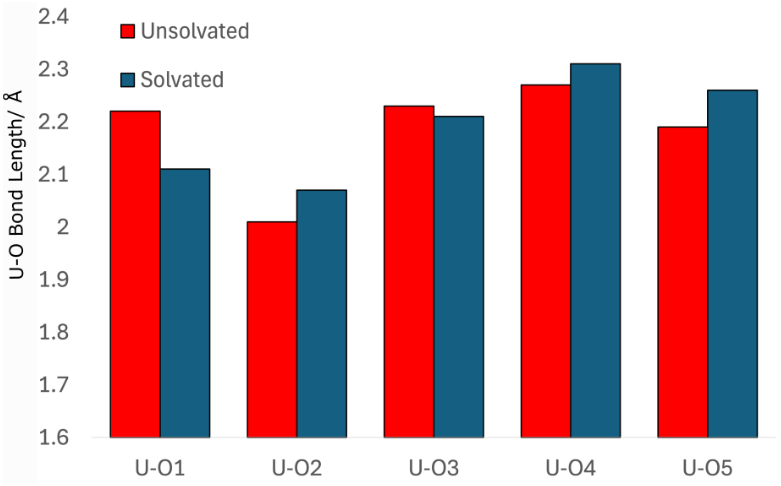

Figure S8: Bar chart comparing U-O bond lengths pre- and post-solvation for U(IV) incorporation at the surface of [010] goethite with a separated iron vacancy.

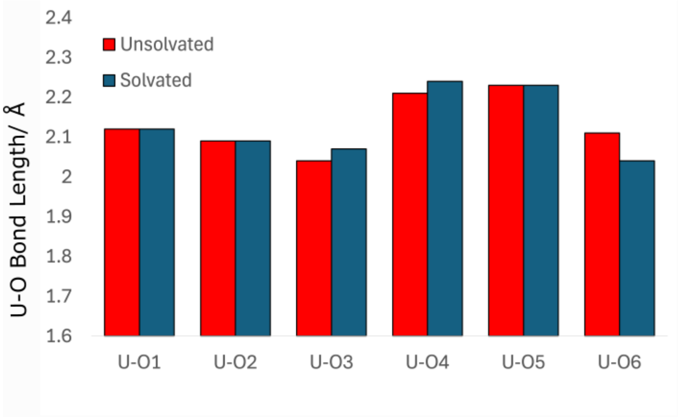

Figure S9: Bar chart comparing U-O bond lengths pre- and post-solvation for U(VI) incorporation and subsequent reduction to U(V) at the near-surface of [010] goethite with a separated iron vacancy.

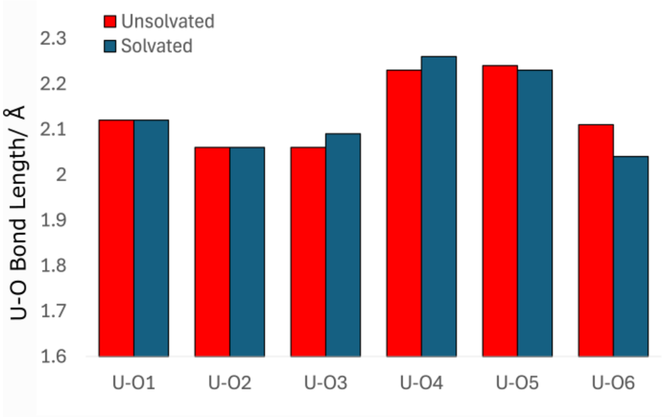

Figure S10: Bar chart comparing U-O bond lengths pre- and post-solvation for U(V) at the near-surface of [010] goethite with a separated iron vacancy.

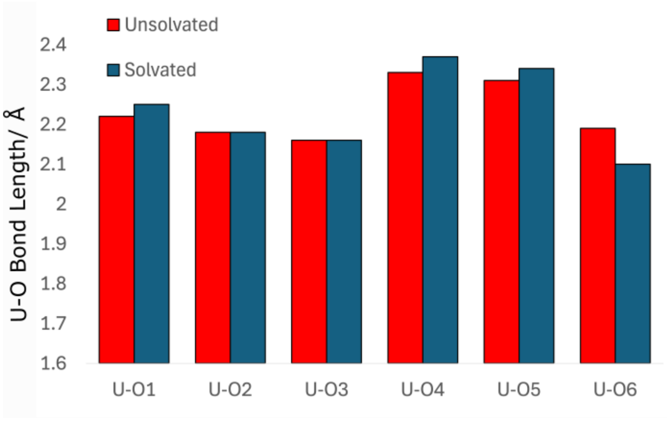

Figure S11: Bar chart comparing U-O bond lengths pre- and post-solvation for U(IV) incorporation at the near-surface of [010] goethite with a separated iron vacancy.

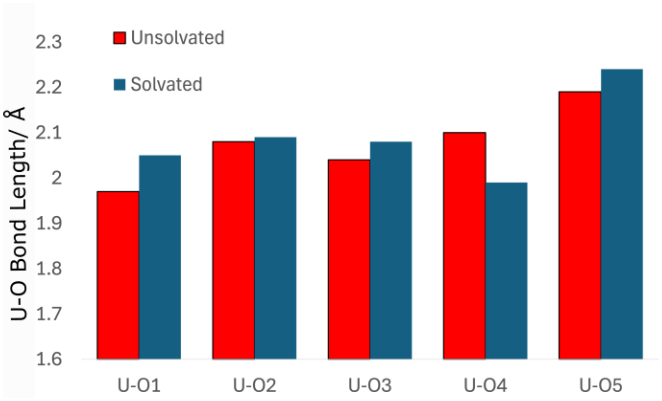

Figure S12: Bar chart comparing U-O bond lengths pre- and post-solvation for U(VI) incorporation and subsequent reduction to U(V) at the surface of [010] goethite with no iron vacancy.

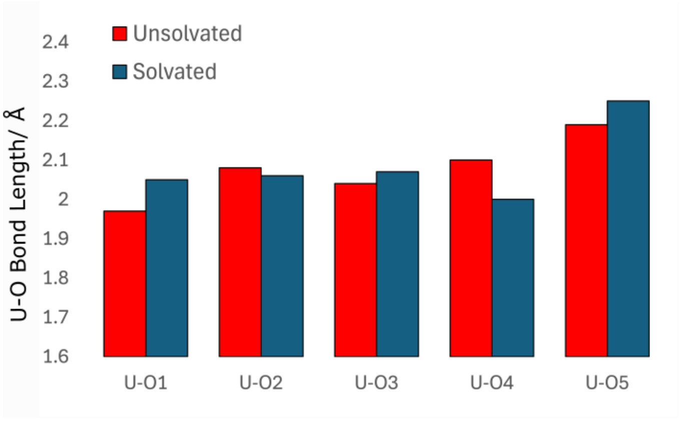

Figure S13: Bar chart comparing U-O bond lengths pre- and post-solvation for U(V) at the surface of [010] goethite with no iron vacancy.

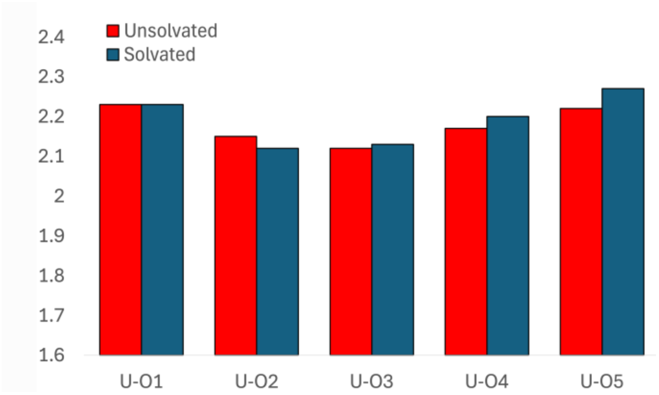

Figure S14: Bar chart comparing U-O bond lengths pre- and post-solvation for U(IV) incorporation at the surface of [010] goethite with no iron vacancy.

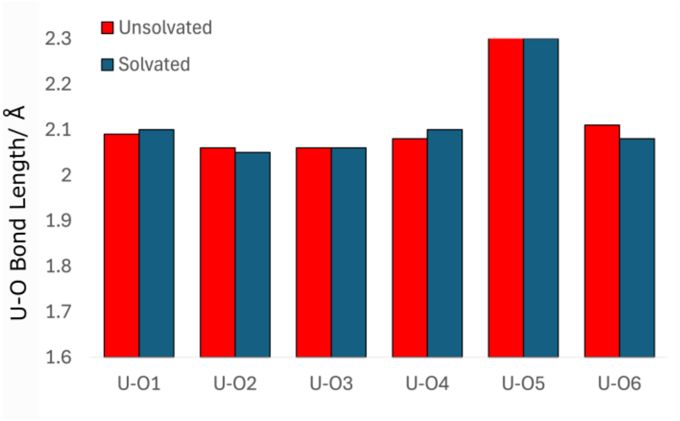

Figure S15: Bar chart comparing U-O bond lengths pre- and post-solvation for U(VI) incorporation and subsequent reduction to U(V) at the near-surface of [010] goethite with no iron vacancy.

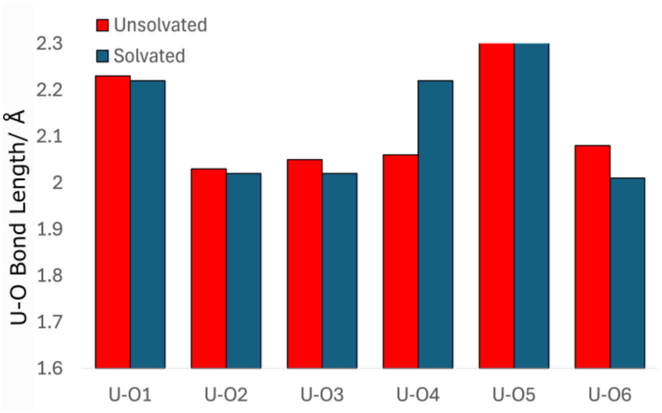

Figure S16: Bar chart comparing U-O bond lengths pre- and post-solvation for U(V) at the near-surface of [010] goethite with no iron vacancy.

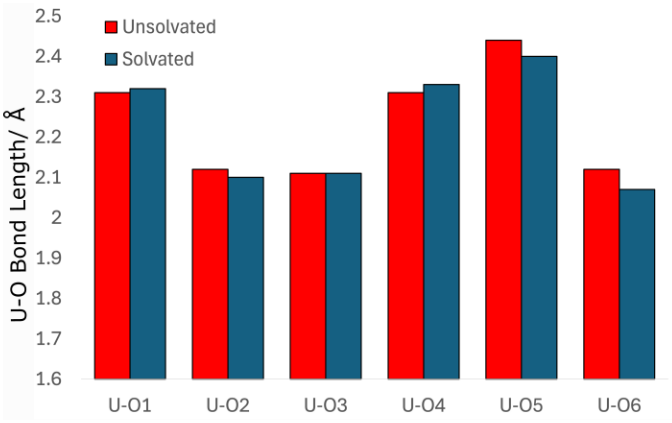

Figure S17: Bar chart comparing U-O bond lengths pre- and post-solvation for U(IV) incorporation at the near- surface of [010] goethite with no iron vacancy.

Section 6: 5f MO imaging

To further verify the oxidation state of the incorporated uranium species, the 5f-based MOs were visualised and Mulliken analysis used to determine the percentage contribution of AOs to the MOs. If U(VI) were incorporated, then no 5f MO was be imaged as there would not be an unpaired electron. For U(V) incorporated species then a singular 5f-based MO should be found and for U(IV) two separate such MOs should be found. Images were visualised in VESTA with an isovalue level of 0.05.

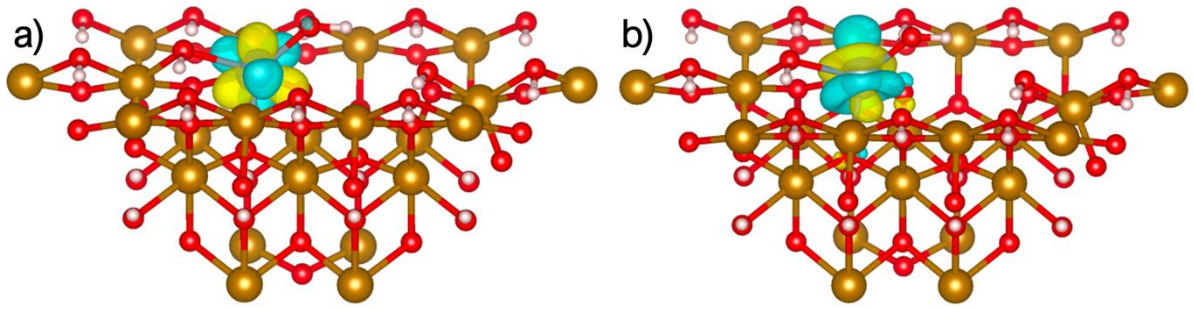

Figure S18: 5f-based MO for U(IV) when incorporated at the surface with an adjacent iron vacancy. a) 5f AO percentage contribution of 96.9% b) 5f AO percentage contribution of 87.1%.

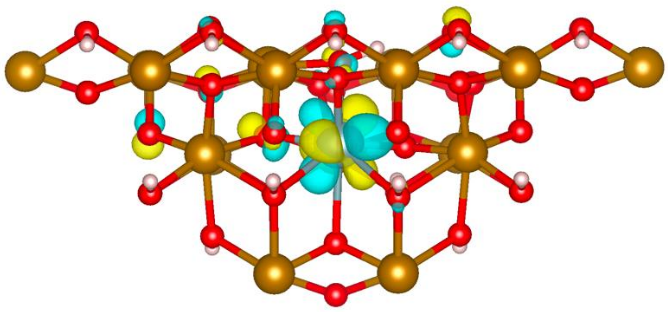

Figure S19: 5f-based MO for U(V) when incorporated U(VI) was reduced to U(V) at the near-surface with an adjacent iron vacancy. 5f AO percentage contribution of 63.8%.

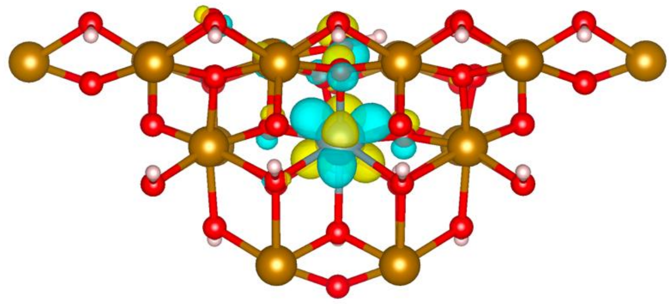

Figure S20: 5f-based MO for U(V) when incorporated at the near-surface with an adjacent iron vacancy. 5f AO percentage contribution of 71.9%

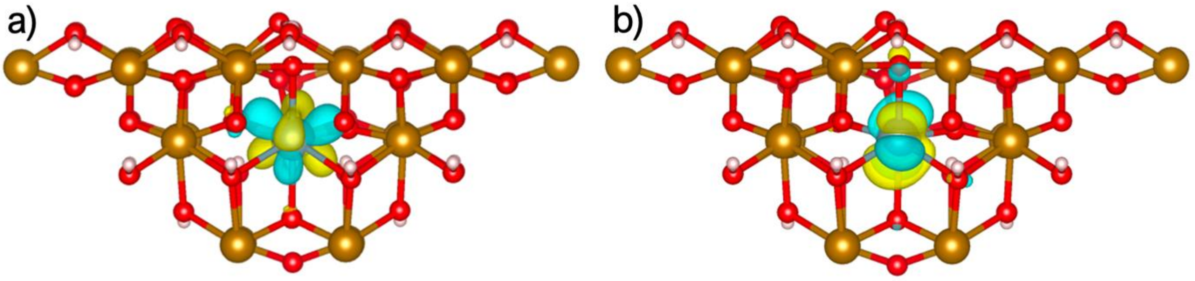

Figure S21: 5f-based MO for U(IV) when incorporated at the near-surface with an adjacent iron vacancy. a) 5f AO percentage contribution of 94.4% b) 5f AO percentage contribution of 93.5%

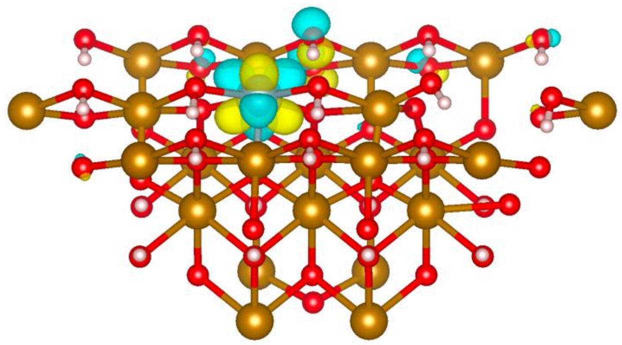

Figure S22: 5f-based MO for U(V) when incorporated U(VI) was reduced to U(V) when incorporated at the surface with a separated iron vacancy. 5f AO percentage contribution of 67.2%

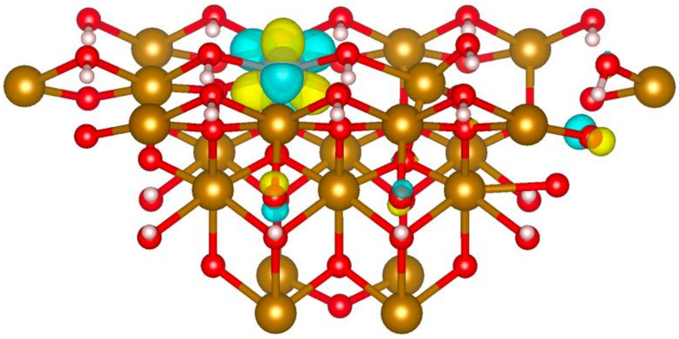

Figure S23: 5f-based MO for U(V) when incorporated at the surface with a separated iron vacancy. 5f AO percentage contribution of 83.6%

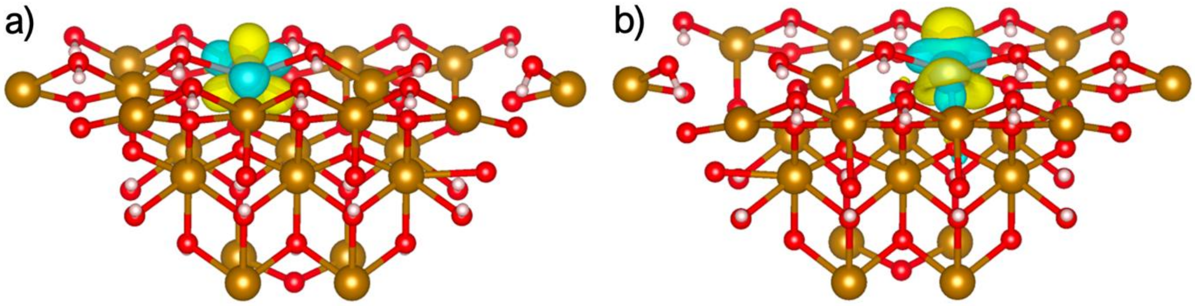

Figure S24: 5f-based MO for U(IV) when incorporated at the surface with a separated iron vacancy. a) 5f AO percentage contribution of 95.6% b) 5f AO percentage contribution of 85.3%

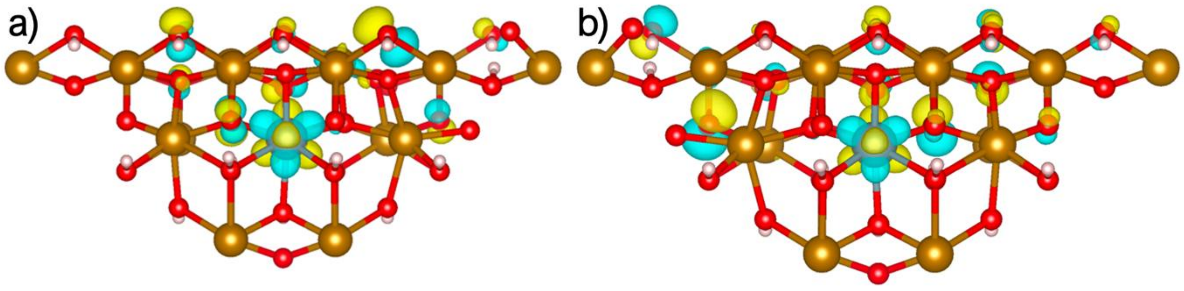

Figure S25: 5f-based MO for U(V) when incorporated U(VI) was reduced to U(V) when incorporated at the near-surface with a separated iron vacancy. a) 5f AO percentage contribution of 32.4% b) 5f AO percentage contribution of 20.5%

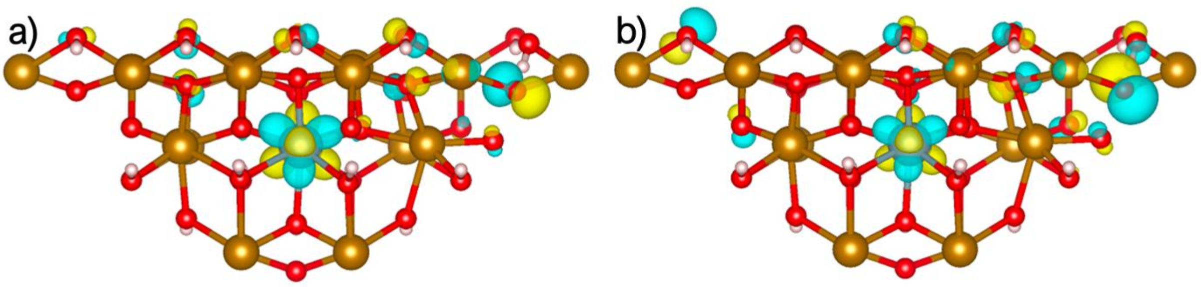

Figure S26: 5f-based MO for U(V) when incorporated at the near-surface with a separated iron vacancy. a) 5f AO percentage contribution of 49.1% b) 5f AO percentage contribution of 24.8%

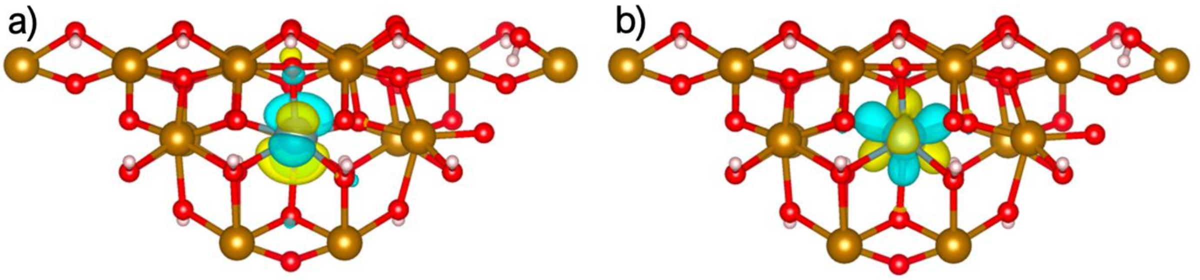

Figure S27: 5f-based MO for U(IV) when incorporated at the near-surface with a separated iron vacancy. a) 5f AO percentage contribution of 92.4% b) 5f AO percentage contribution of 94.5%

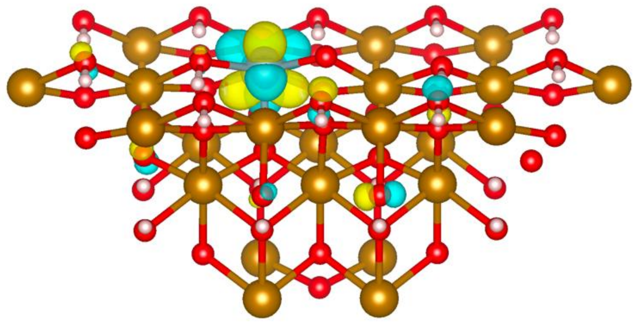

Figure S28: 5f-based MO for U(V) when incorporated U(VI) was reduced to U(V) when incorporated at the surface without an iron vacancy. 5f AO percentage contribution of 78.8%

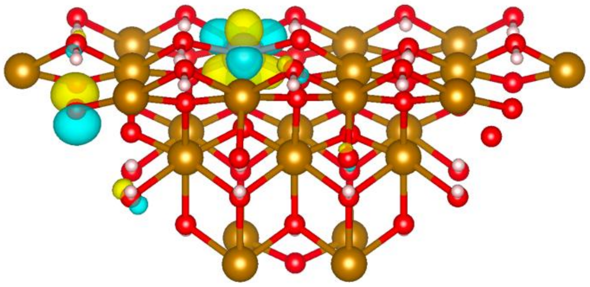

Figure S29: 5f-based MO for U(V) when incorporated at the surface without an iron vacancy. 5f AO percentage contribution of 67.0%

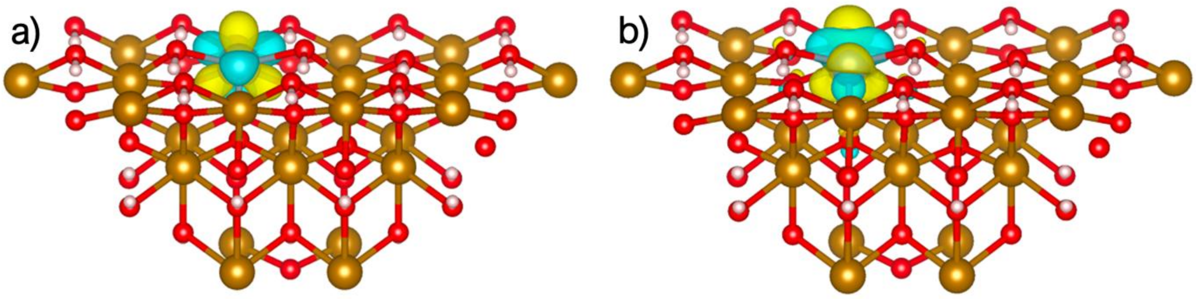

Figure S30: 5f-based MO for U(IV) when incorporated at the surface without an iron vacancy. a) 5f AO percentage contribution of 95.8% b) 5f AO percentage contribution of 86.3%

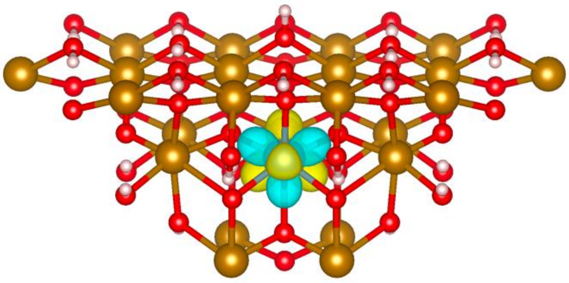

Figure S31: 5f-based MO for U(V) when incorporated U(VI) was reduced to U(V) when incorporated at the near-surface without an iron vacancy. 5f AO percentage contribution of 95.5%

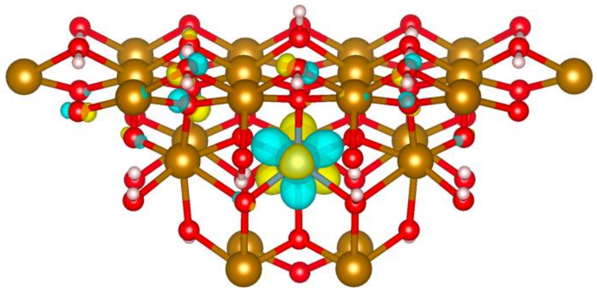

Figure S32: 5f-based MO for U(V) when incorporated at the near-surface without an iron vacancy. 5f AO percentage contribution of 70.5%

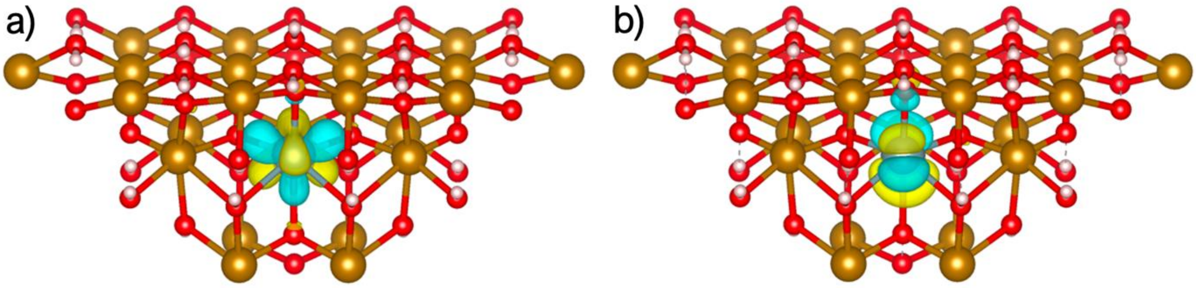

Figure S33: 5f-based MO for U(IV) when incorporated at the near-surface without an iron vacancy. a) 5f AO percentage contribution of 92.7% b) 5f AO percentage contribution of 90.0%

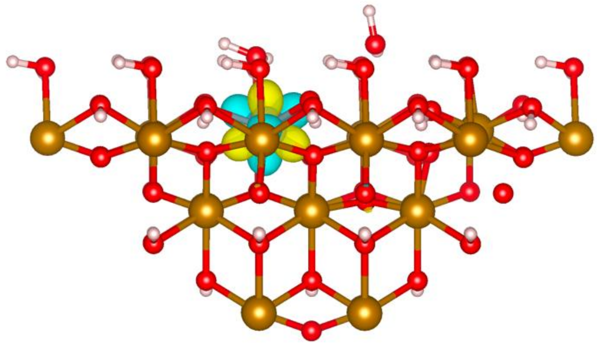

Figure S34: 5f-based MO for U(V) when incorporated U(VI) was reduced to U(V) when incorporated at the surface with an adjacent iron vacancy in the solvated model. 5f AO percentage contribution of 93.3%

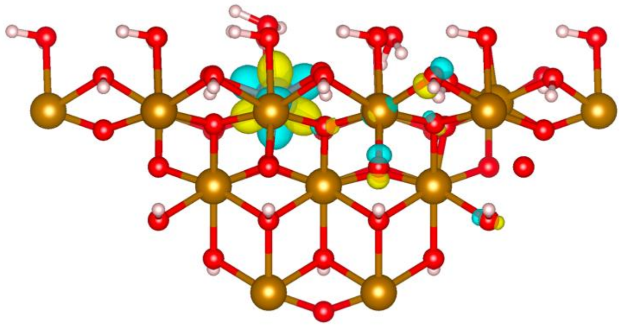

Figure S35: 5f-based MO for U(V) when incorporated at the surface with an adjacent iron vacancy in the solvated model. 5f AO percentage contribution of 84.6%

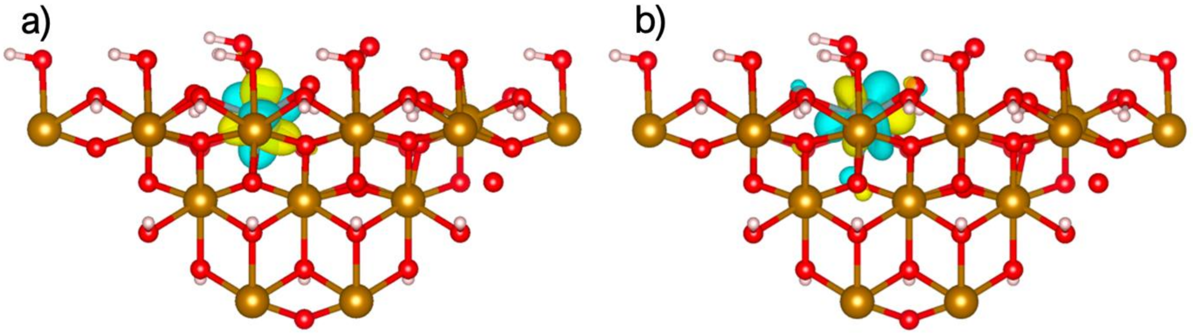

Figure S36: 5f-based MO for U(IV) when incorporated at the surface with an adjacent iron vacancy in the solvated model. a) 5f AO percentage contribution of 89.4% b) 5f AO percentage contribution of 95.9%.

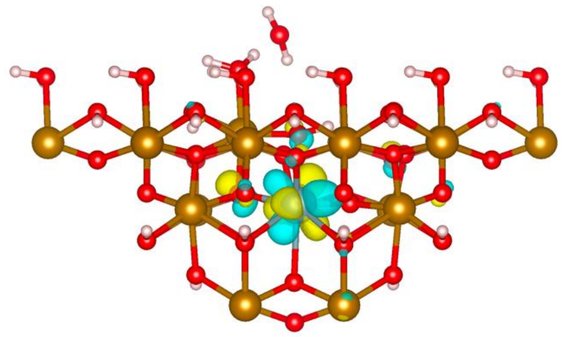

Figure S37: 5f-based MO for U(V) when incorporated U(VI) was reduced to U(V) at the near-surface with an adjacent iron vacancy in the solvated model. 5f AO percentage contribution of 69.4%.

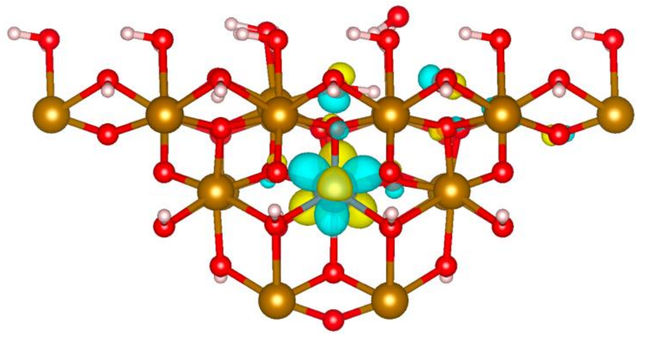

Figure S38: 5f-based MO for U(V) when incorporated at the near-surface with an adjacent iron vacancy in the solvated model. 5f AO percentage contribution of 78.0%.

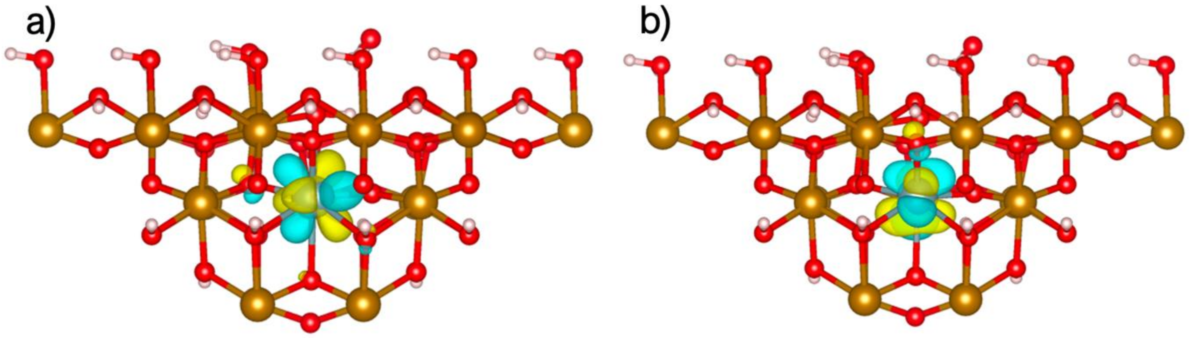

Figure S39: 5f-based MO for U(IV) when incorporated at the near-surface with an adjacent iron vacancy in the solvated model. a) 5f AO percentage contribution of 91.4% b) 5f AO percentage contribution of 90.1%

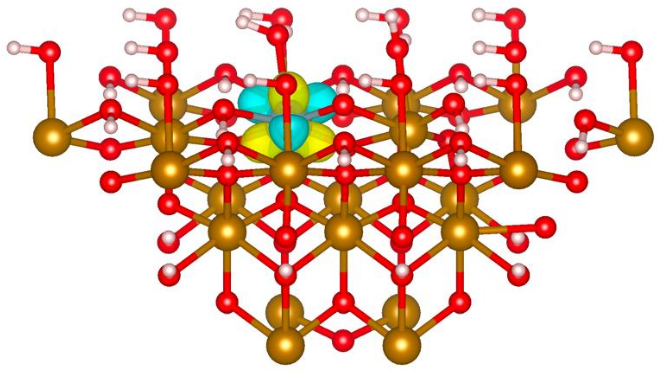

Figure S40: 5f-based MO for U(V) when incorporated U(VI) was reduced to U(V) when incorporated at the surface with a separated iron vacancy in the solvated model. 5f AO percentage contribution of 96.9%

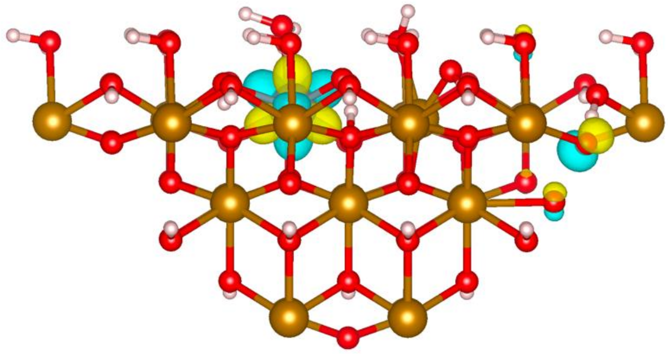

Figure S41: 5f-based MO for U(V) when incorporated at the surface with a separated iron vacancy in the solvated model.   5f AO percentage contribution of 82.7%

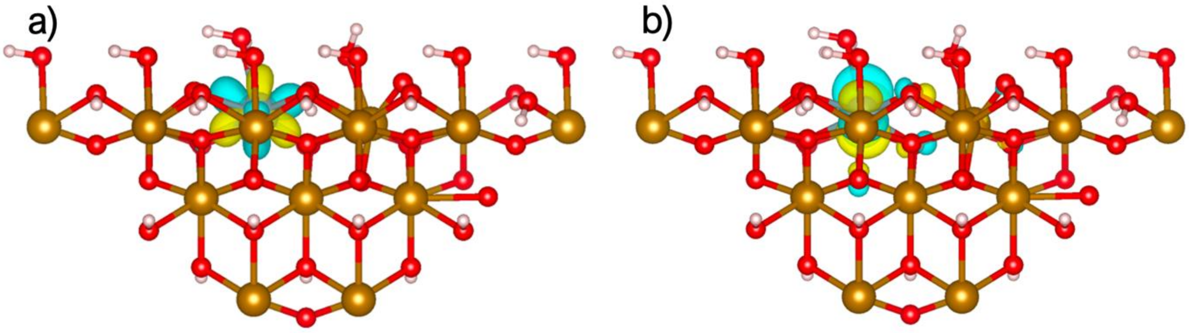

Figure S42: 5f-based MO for U(IV) when incorporated at the surface with a separated iron vacancy in the solvated model. a) 5f AO percentage contribution of 95.5% b) 5f AO percentage contribution of 86.4%

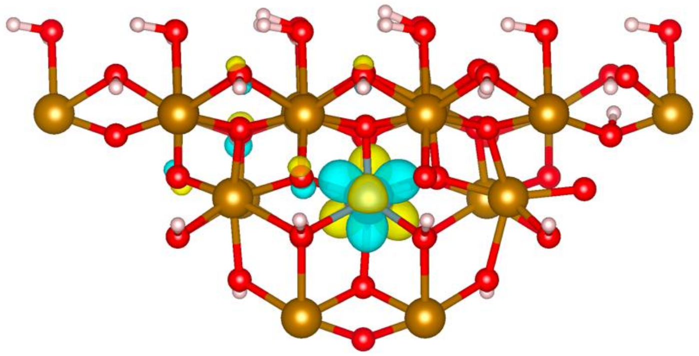

Figure S43: 5f-based MO for U(V) when incorporated U(VI) was reduced to U(V) when incorporated at the near-surface with a separated iron vacancy in the solvated model. 5f AO percentage contribution of 82.2%

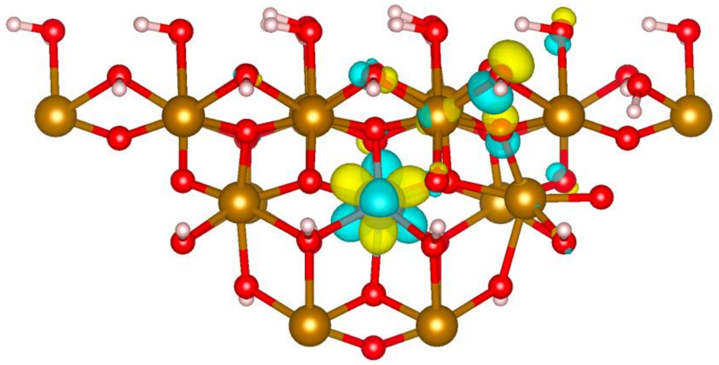

Figure S44: 5f-based MO for U(V) when incorporated at the near-surface with a separated iron vacancy in the solvated model. 5f AO percentage contribution of 58.2%

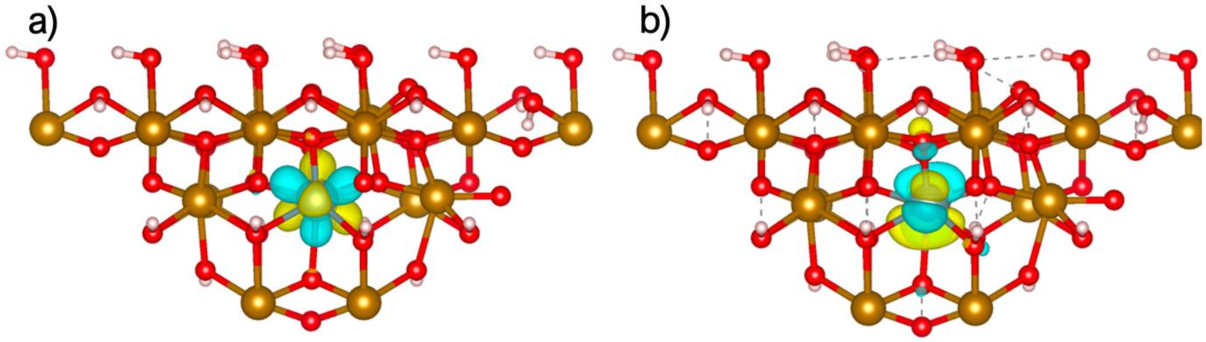

Figure S45: 5f-based MO for U(IV) when incorporated at the near-surface with a separated iron vacancy in the solvated model. a) 5f AO percentage contribution of 95.0% b) 5f AO percentage contribution of 89.5%

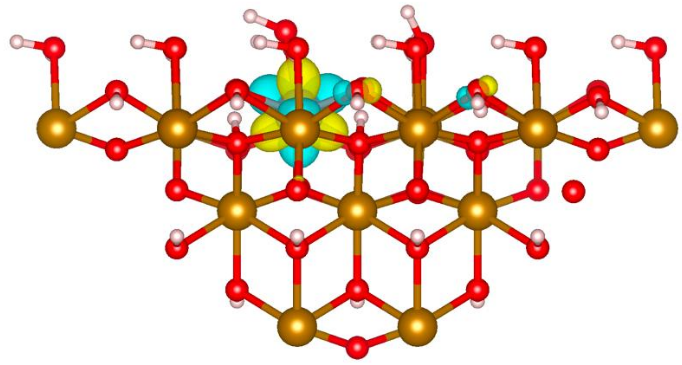

Figure S46: 5f-based MO for U(V) when incorporated U(VI) was reduced to U(V) when incorporated at the surface without an iron vacancy in the solvated model. 5f AO percentage contribution of 91.3%

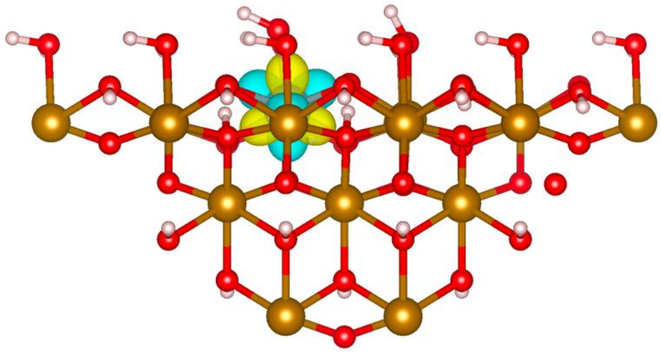

Figure S47: 5f-based MO for U(V) when incorporated at the surface without an iron vacancy in the solvated model. 5f AO percentage contribution of 97.5%

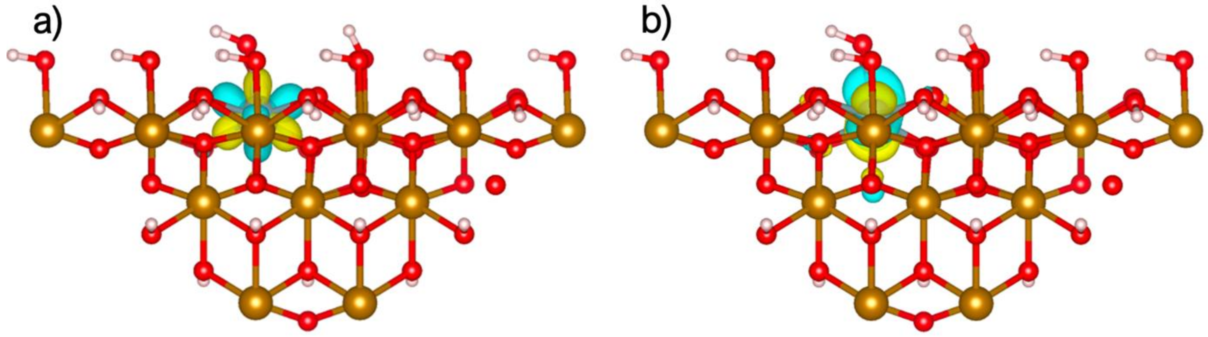

Figure S48: 5f-based MO for U(IV) when incorporated at the surface without an iron vacancy. in the solvated model a) 5f AO percentage contribution of 91.7% b) 5f AO percentage contribution of 95.2%

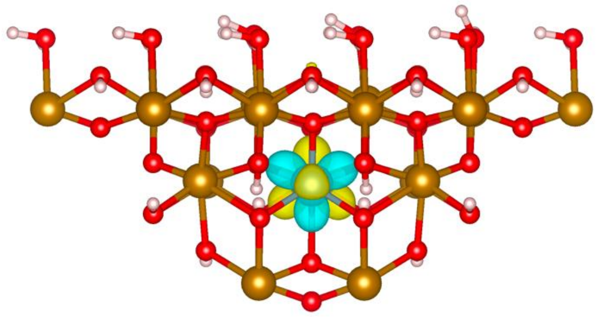

Figure S49: 5f-based MO for U(V) when incorporated U(VI) was reduced to U(V) when incorporated at the near-surface without an iron vacancy in the solvated model. 5f AO percentage contribution of 95.5%.

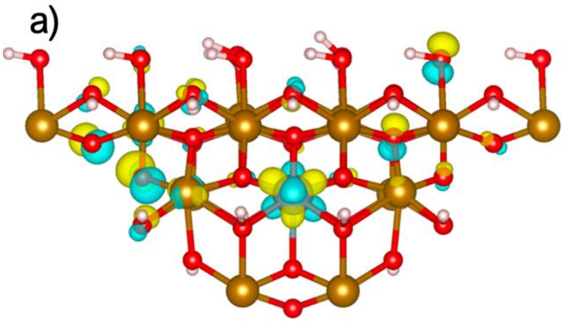

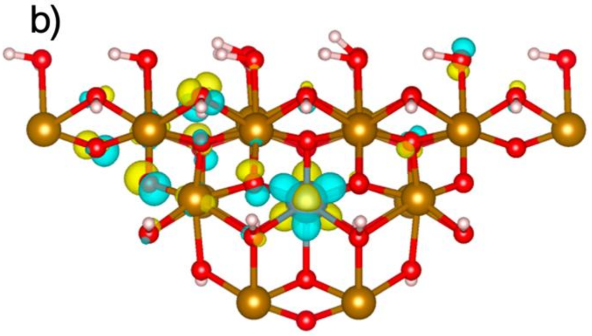

Figure S50: 5f-based MO for U(V) when incorporated at the near-surface without an iron vacancy in the solvated model. a) 5f AO percentage contribution of 22.3% b) 5f AO percentage contribution of 33.3%

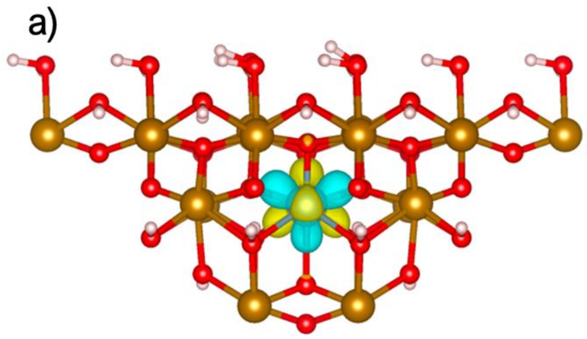

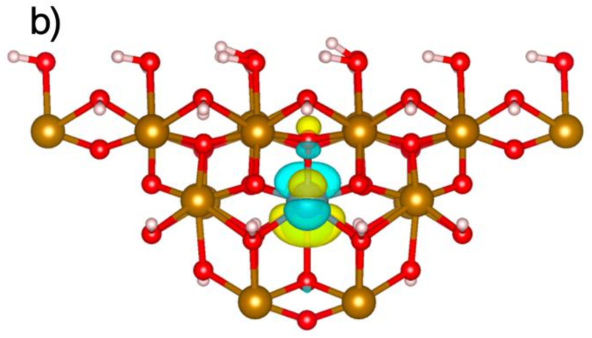

Figure S51: 5f-based MO for U(IV) when incorporated at the near-surface without an iron vacancy in the solvated model a) 5f AO percentage contribution of 94.8% b) 5f AO percentage contribution of 88.0%

<u>Section 7: Changes in spin density compared to equivalent unincorporated</u>

For the models where U(VI) is reduced to U(V), changes in the spin density and charge were calculated to determine which atoms were participating in the reduction of U(VI) to U(V). To do so, models with just the iron vacancies were calculated to determine the spin density and charge for each atom as well as for the models were U(VI) was incorporated. As can be seen in Tables S27-36, oxygens surrounding the incorporated uranium species see the largest change in spin density and charge. The oxo-oxygens were seen to have a larger electron transfer to the uranium than hydroxyl oxygens. The numbering of atoms is consistent between models and represents the number found in the coord files, where the numbering of key atoms can be seen in Figures S52 and S53. Atoms 96 to 101, which are included in the solvated models (Tables S16-S21) are the unfixed water atoms above positions 1 and 18.

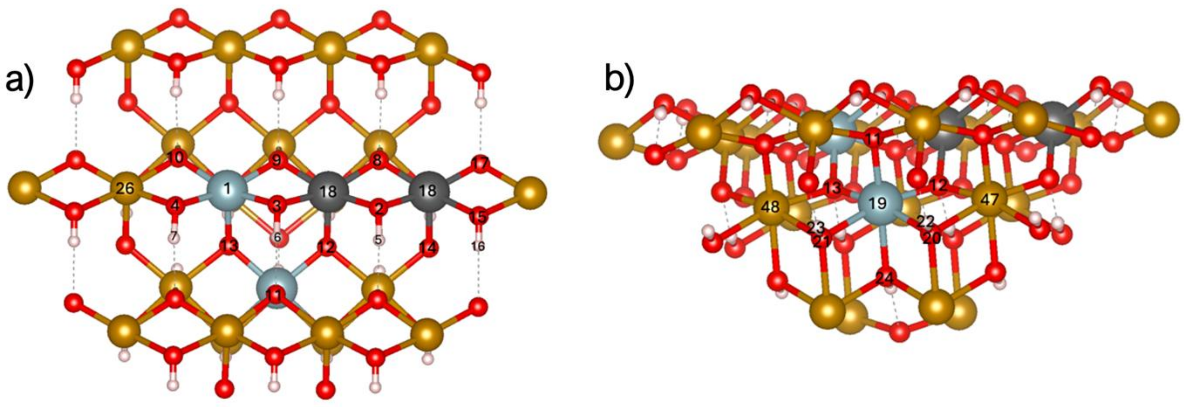

Figure S52: Numbering of important atoms found from changes in the spin density and charge, post U(VI) reduction to U(V) for models where there is an iron vacancy. Silver atoms are used to denote where uranium species were incorporated and black/dark silver atoms denote possible iron vacancy positions. Gold spheres represent iron atoms, red spheres are used for oxygen atoms and white spheres are for hydrogen atoms. a) Shows the top side of the surface and b) Shows a side on profile.

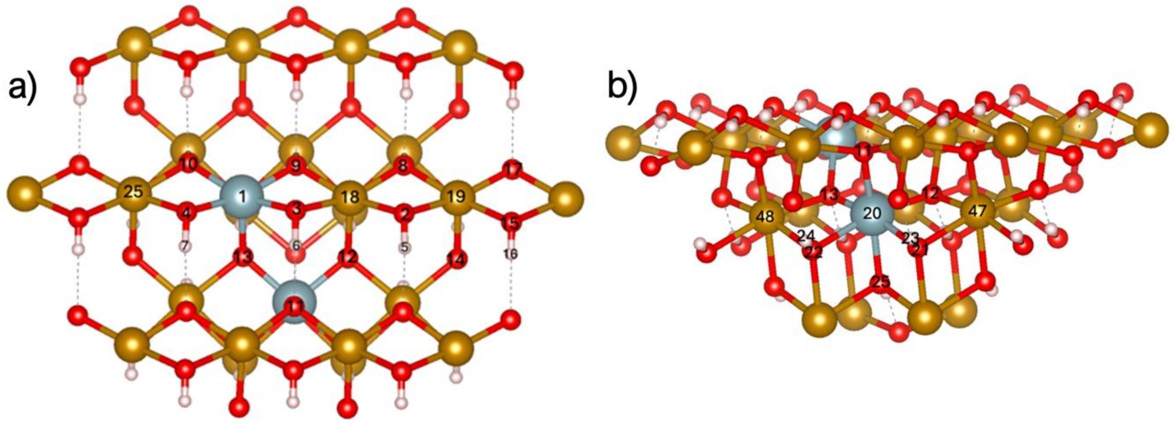

Figure S53: Numbering of important atoms found from changes in the spin density and charge, post U(VI) reduction to U(V) for models where there is not an iron vacancy. Silver atoms are used to denote where uranium species were incorporated. Gold spheres represent iron atoms, red spheres are used for oxygen atoms and white spheres are for hydrogen atoms. a) Shows the top side of the surface and b) Shows a side on profile.

Table S27: Change in number of unpaired electrons and charge for certain atoms after addition of uranium species for when U(VI) species are incorporated at the surface with an adjacent vacancy in the unsolvated [010] goethite model. The first 25 atoms in the embedded cluster have been compared as this is where significant changes in spin density and charge were calculated. f is used to denote a fixed atom in the calculation, where the geometry is unable to change during the optimisation process.

		Changes in spin density and charge compared between no U(VI) model and U(VI) incorporated		Changes in spin density and charge compared between no U(VI) model and U(VI) reduced to U(V)		Changes in spin density and charge compared between U(VI) model and U(VI) reduced to U(V)	
atoms	Type and fixing	Change in spin density	Change in charge	change sum	change charge	Changes in spin density	Changes in charge
1	U					-1.164	0.010
2	O	0.126	0.130	-0.534	-0.480	0.661	0.611
3	O	0.777	-0.072	0.732	0.162	0.045	-0.234
4	O	0.139	-0.192	0.117	-0.156	0.022	-0.036
5	H	0.000	-0.024	0.017	0.019	-0.017	-0.044
6	H	-0.017	-0.050	-0.007	-0.031	-0.009	-0.019
7	H	0.002	-0.039	0.003	-0.035	-0.001	-0.003
8	O	-0.006	-0.006	0.021	0.016	-0.027	-0.022
9	O	0.791	-0.135	0.707	-0.022	0.085	-0.113

10	O	0.306	-0.272	0.231	-0.196	0.075	-0.076
11	O	0.219	-0.078	0.024	-0.003	0.195	-0.075
12	O	0.124	-0.124	-0.005	-0.013	-0.119	-0.111
13	O	0.234	-0.204	0.185	-0.172	0.049	-0.032
14	O	-0.019	-0.016	-0.002	-0.006	-0.017	-0.009
15	O	-0.008	0.010	-0.295	-0.044	0.287	0.054
16	H	0.003	-0.009	0.000	0.000	0.004	-0.008
17	O f	-0.049	-0.044	-0.071	-0.046	0.022	0.002
18	F e	-0.071	-0.066	-0.011	0.015	-0.060	-0.081
19	Fe f	0.209	0.158	0.022	-0.005	0.187	0.163
20	O f	-0.020	-0.011	-0.040	-0.018	0.020	0.007
21	O f	-0.011	-0.007	-0.037	-0.015	0.026	0.008
22	O f	0.000	-0.007	0.001	-0.010	-0.001	0.004
23	H f	0.001	-0.009	0.001	-0.012	0.000	0.003
24	H f	-0.051	-0.026	-0.026	-0.009	-0.026	-0.017
25	Fe f	0.035	-0.017	0.016	-0.029	0.019	0.011

Table S28: Change in number of unpaired electrons and charge for each atom after addition of uranium species for when U(VI) species are incorporated at the near-surface with an adjacent vacancy in the unsolvated [010] goethite model. The first 25 atoms and any other unfixed atoms in the embedded cluster have been compared as this is where significant changes in spin density and charge were calculated. f is used to denote a fixed atom in the calculation, where the geometry is unable to change during the optimisation process.

		Changes in spin density and charge compared between no U(VI) model and U(VI) incorporated		Changes in spin density and charge compared between no U(VI) model and U(VI) reduced to U(V)		Changes in spin density and charge compared between U(VI) model and U(VI) reduced to U(V)	
atoms	Type and fixing	Change in spin density	Change in charge	change sum	change charge	Changes in spin density	Changes in charge
1	Fe	-0.067	-0.130	-0.075	-0.138	0.008	0.008
2	O f	0.220	0.191	0.395	0.316	-0.175	-0.125
3	O	0.163	-0.085	0.180	-0.070	-0.016	-0.015
4	O	0.004	0.101	-0.017	0.097	0.020	0.004
5	H f	-0.006	-0.006	-0.014	-0.008	0.007	0.001
6	H	0.018	-0.061	0.018	-0.078	0.000	0.017
7	H	0.001	-0.108	0.002	-0.109	-0.002	0.002
8	O f	-0.045	-0.048	-0.114	-0.113	0.069	0.065
9	O	0.740	0.678	0.763	0.694	-0.023	-0.016

10	O f	-0.013	0.008	-0.018	0.004	0.004	0.004
11	O	0.129	-0.330	0.183	-0.288	-0.054	-0.042
12	O	0.666	-0.336	0.592	-0.191	0.075	-0.145
13	O	0.277	-0.334	0.269	-0.249	0.007	-0.085
14	O f	-0.047	-0.039	-0.035	-0.037	-0.012	-0.002
15	O f	-0.043	-0.031	-0.010	-0.009	-0.033	-0.023
16	H f	0.002	-0.001	0.000	-0.002	0.002	0.001
17	O f	0.003	0.004	-0.065	-0.043	0.069	0.047
18	Fe f	-0.063	-0.059	-0.053	-0.069	-0.011	0.010
19	U					-1.056	0.080
20	O	0.071	-0.168	-0.012	-0.135	0.083	-0.032
21	O	0.094	-0.317	-0.011	-0.126	0.106	-0.191
22	O	0.001	-0.076	0.002	-0.080	-0.001	0.003
23	H	0.002	-0.056	0.004	-0.077	-0.002	0.021
24	H	0.081	-0.088	0.012	-0.051	0.069	-0.037
25	Fe f	-0.053	-0.065	-0.053	-0.061	0.001	-0.004
45	Fe	-0.489	-0.638	-0.483	-0.632	-0.007	-0.006
46	Fe	0.009	0.000	0.014	0.005	-0.005	-0.005

Table S29: Change in number of unpaired electrons and charge for each atom after addition of uranium species for when U(VI) species are incorporated at the surface with a separated vacancy in the unsolvated [010] goethite model. The first 25 atoms and any other unfixed atoms in the embedded cluster have been compared as this is where significant changes in spin density and charge were calculated. f is used to denote a fixed atom in the calculation, where the geometry is unable to change during the optimisation process.

		Changes in spin density and charge compared between no U(VI) model and U(VI) incorporated	
atoms	Type and fixing	Change in spin density	Change in charge
1	U		
2	O	-0.087	-0.079
3	O	0.143	-0.186
4	O	0.130	-0.189
5	H	-0.002	-0.015
6	H	0.001	-0.033
7	H	0.003	-0.037
8	O	0.002	-0.008
9	O	0.301	-0.317
10	O	0.245	-0.244
11	O	0.084	-0.001
12	O	-0.019	-0.024
13	O	0.241	-0.192

14	O	-0.262	-0.232
15	O	0.022	0.014
16	H	-0.001	0.021
17	O f	0.012	0.019
18	F e	0.098	0.036
19	Fe f	0.015	-0.017
20	O f	-0.026	-0.016
21	O f	-0.015	-0.010
22	O f	0.000	-0.005
23	H f	0.001	-0.008
24	H f	0.011	0.011
25	Fe f	0.057	0.001

Table S30: Change in number of unpaired electrons and charge for each atom after addition of uranium species for when U(VI) species are incorporated at the near-surface with a separated vacancy in the unsolvated [010] goethite model. The first 25 atoms and any other unfixed atoms in the embedded cluster have been compared as this is where significant changes in spin density and charge were calculated. f is used to denote a fixed atom in the calculation, where the geometry is unable to change during the optimisation process.

		Changes in spin density and charge compared between no U(VI) model and when U(VI) is initially incorporated	
atoms	Type and fixing	Change in spin density	Change in charge
1	Fe f	0.006	-0.027
2	O	0.053	0.033
3	O f	0.010	-0.032
4	O f	-0.007	-0.016
5	H	-0.002	-0.004
6	H f	0.000	-0.013
7	H f	0.000	-0.005
8	O	-0.013	-0.018
9	O f	0.020	-0.004
10	O f	-0.011	-0.012
11	O	0.219	-0.260
12	O	0.233	-0.292
13	O	0.265	-0.316

14	O	-0.022	-0.025
15	O	-0.631	-0.305
16	H	-0.007	-0.003
17	O f	-0.093	-0.086
18	Fe	-0.001	-0.044
19	U		
20	O	0.158	-0.260
21	O	0.102	-0.232
22	H	0.001	-0.031
23	H	0.000	-0.032
24	O	0.099	-0.183
25	Fe f	-0.028	-0.028
47	Fe f	0.0335	-0.0081

Table S31: Change in number of unpaired electrons and charge for each atom after addition of uranium species for when U(VI) species are incorporated at the surface without an iron vacancy in the unsolvated [010] goethite model. The first 25 atoms and any other unfixed atoms in the embedded cluster have been compared as this is where significant changes in spin density and charge were calculated. f is used to denote a fixed atom in the calculation, where the geometry is unable to change during the optimisation process.

		Changes in spin density and charge compared between no U(VI) model and U(VI) incorporated	
atoms	Type and fixing	Change in spin density	Change in charge
1	U		
2	O	-0.038	-0.007
3	O	0.103	-0.228
4	O	0.158	-0.187
5	H	-0.001	-0.010
6	H	-0.003	-0.028
7	H	0.002	-0.031
8	O	0.011	0.009
9	O	0.238	-0.233
10	O	0.284	-0.249
11	O	0.199	-0.077
12	O	0.038	0.022
13	O	0.190	-0.179

14	O	0.010	0.034
15	O	0.046	-0.036
16	H	0.003	0.005
17	O f	-0.017	-0.011
18	Fe	0.052	-0.003
19	Fe f	-0.010	-0.021
20	Fe f	0.003	-0.016
21	O f	-0.009	-0.005
22	O f	0.008	0.001
23	H f	0.000	-0.005
24	H f	0.000	-0.005
25	O f	-0.001	0.009
47	Fe f	0.0335	-0.0081

Table S32: Change in number of unpaired electrons and charge for each atom after addition of uranium species for when U(VI) species are incorporated at the surface with an adjacent vacancy in the solvated [010] goethite model. The first 25 atoms and any other unfixed atoms in the embedded cluster have been compared as this is where significant changes in spin density and charge were calculated. f is used to denote a fixed atom in the calculation, where the geometry is unable to change during the optimisation process.

		Changes in spin density and charge compared between no U(VI) model and U(VI) incorporated		Changes in spin density and charge compared between no U(VI) model and U(VI) reduced to U(V)		Changes in spin density and charge compared between U(VI) model and U(VI) reduced to U(V)	
atoms	Type and fixing	Change in spin density	Change in charge	change sum	change charge	Changes in spin density	Changes in charge
1	U					1.157	0.027
2	O	0.029	0.028	0.016	0.019	-0.012	-0.009
3	O	0.361	-0.374	0.323	-0.153	-0.039	0.221
4	O	0.138	-0.221	0.112	-0.185	-0.026	0.036
5	H	-0.002	-0.012	-0.002	-0.014	0.000	-0.002
6	H	-0.006	-0.041	-0.001	-0.035	0.005	0.007
7	H	0.001	-0.029	0.002	-0.027	0.000	0.002
8	O	0.088	0.083	0.084	0.079	-0.004	-0.004
9	O	1.007	0.102	0.926	0.190	-0.081	0.087

10	O	0.313	-0.294	0.227	-0.220	-0.086	0.074
11	O	0.227	-0.072	0.053	-0.006	-0.174	0.066
12	O	-0.025	-0.034	-0.025	-0.040	0.000	-0.005
13	O	0.250	-0.241	0.199	-0.199	-0.051	0.042
14	O	-0.300	-0.240	-0.294	-0.232	0.006	0.008
15	O	0.117	0.025	0.118	0.024	0.001	0.000
16	H	0.000	-0.006	0.000	-0.005	0.000	0.000
17	O f	-0.009	-0.009	-0.005	-0.007	0.003	0.002
18	Fe	-0.068	-0.040	-0.062	-0.034	0.006	0.006
19	Fe f	0.033	0.013	0.034	0.010	0.001	-0.003
20	O f	-0.002	-0.006	-0.046	-0.025	-0.044	-0.019
21	O f	0.010	-0.002	-0.043	-0.025	-0.053	-0.023
22	H f	0.000	-0.001	0.002	-0.007	0.002	-0.007
23	H f	0.001	-0.003	0.001	-0.010	0.000	-0.007
24	O f	0.008	0.013	-0.040	-0.019	-0.048	-0.032
25	Fe f	0.027	-0.022	0.006	-0.036	-0.021	-0.015
97	O	-0.001	-0.009	-0.001	-0.012	0.000	-0.003
98	H	-0.001	0.028	-0.001	0.027	0.000	0.000
99	H	-0.001	-0.030	-0.001	-0.030	0.000	0.000
100	O	0.042	-0.085	0.038	-0.081	-0.004	0.004
101	H	-0.001	-0.016	0.000	-0.019	0.001	-0.003
102	H	0.002	-0.051	0.001	-0.049	0.000	0.002

Table S33: Change in number of unpaired electrons and charge for each atom after addition of uranium species for when U(VI) species are incorporated at the near-surface with an adjacent vacancy in the solvated [010] goethite model. The first 25 atoms and any other unfixed atoms in the embedded cluster have been compared as this is where significant changes in spin density and charge were calculated. f is used to denote a fixed atom in the calculation, where the geometry is unable to change during the optimisation process.

		Changes in spin density and charge compared between no U(VI) model and U(VI) incorporated		Changes in spin density and charge compared between no U(VI) model and U(VI) reduced to U(V)		Changes in spin density and charge compared between U(VI) model and U(VI) reduced to U(V)	
atoms	Type and fixing	Change in spin density	Change in charge	change sum	change charge	Changes in spin density	Changes in charge
1	U					-0.020	-0.025
2	O	-0.066	-0.040	0.025	0.021	0.091	0.061
3	O	-0.067	-0.095	-0.001	-0.157	0.066	-0.061
4	O	-0.014	-0.010	-0.032	-0.016	-0.019	-0.006
5	H	0.000	0.010	-0.004	0.009	-0.005	-0.001
6	H	0.000	-0.022	0.000	-0.046	0.000	-0.024
7	H	-0.001	-0.003	-0.001	-0.005	0.000	-0.001
8	O	0.015	0.015	-0.017	-0.015	-0.032	-0.030
9	O	-0.457	-0.405	-0.439	-0.402	0.018	0.003

10	O	-0.012	0.044	-0.020	0.037	-0.008	-0.007
11	O	0.347	-0.272	0.373	-0.223	0.027	0.049
12	O	0.503	-0.459	0.430	-0.322	-0.074	0.138
13	O	0.266	-0.386	0.249	-0.300	-0.017	0.086
14	O	0.529	0.500	0.540	0.505	0.011	0.006
15	O	-0.143	-0.032	-0.121	-0.015	0.022	0.017
16	H	-0.001	-0.008	-0.003	-0.011	-0.001	-0.003
17	O f	0.094	0.287	0.041	0.251	-0.052	-0.035
18	F e	-0.045	-0.037	-0.012	-0.034	0.033	0.003
19	Fe f	4.110	0.879	5.153	0.841	1.043	-0.037
20	O f	0.081	-0.245	-0.021	-0.202	-0.102	0.043
21	O f	0.082	-0.361	-0.028	-0.191	-0.110	0.170
22	O f	-0.004	-0.045	-0.002	-0.046	0.002	-0.002
23	H f	-0.001	-0.024	0.001	-0.045	0.002	-0.021
24	H f	0.074	-0.218	0.008	-0.175	-0.066	0.044
25	Fe f	-0.132	-0.037	-0.129	-0.031	0.003	0.006
45	Fe	-0.172	-0.186	-0.171	-0.184	0.001	0.002
46	Fe	-0.187	-0.227	-0.190	-0.229	-0.003	-0.002
97	o	0.002	-0.063	-0.001	-0.042	-0.003	0.021
98	h	0.000	0.033	-0.001	0.026	0.000	-0.007
99	h	-0.001	0.001	0.002	-0.030	0.003	-0.031
100	o	-0.014	0.025	-0.037	0.077	-0.024	0.053

101	h	0.001	-0.025	0.000	-0.019	-0.001	0.006
102	h	0.000	-0.026	0.001	-0.049	0.001	-0.024

Table S34: Change in number of unpaired electrons and charge for each atom after addition of uranium species for when U(VI) species are incorporated at the surface with a separated vacancy in the solvated [010] goethite model. The first 25 atoms and any other unfixed atoms in the embedded cluster have been compared as this is where significant changes in spin density and charge were calculated. f is used to denote a fixed atom in the calculation, where the geometry is unable to change during the optimisation process.

		Changes in spin density and charge compared between no U(VI) model and U(VI) incorporated	
atoms	Type and fixing	Change in spin density	Change in charge
1	U		
2	O	-0.1685	-0.0980
3	O	0.1028	-0.2730
4	O	0.0960	-0.2010
5	H	-0.0017	-0.0203
6	H	-0.0028	-0.0378
7	H	0.0012	-0.0350
8	O	-0.4119	-0.3836
9	O	0.2660	-0.2963
10	O	0.2293	-0.2756

11	O	0.2091	-0.0552
12	O	0.0402	0.0217
13	O	0.1951	-0.2149
14	O	-0.0052	-0.0025
15	O	-0.0460	-0.0376
16	H	0.0027	0.0033
17	o f	0.0666	0.0743
18	fe	0.0733	0.0176
19	Fe f	-0.0052	-0.0237
20	O f	-0.0089	-0.0080
21	O f	-0.0020	-0.0068
22	H f	0.0007	-0.0043
23	H f	0.0008	-0.0046
24	O f	0.0052	0.0108
25	Fe f	0.0359	-0.0192
96	O	0.015	-0.016
97	H	-0.001	0.002
98	H	0.000	-0.026
99	O	0.031	-0.098
100	H	0.000	-0.015
101	H	0.001	-0.044

Table S35: Change in number of unpaired electrons and charge for each atom after addition of uranium species for when U(VI) species are incorporated at the near-surface with a separated vacancy in the solvated [010] goethite model. The first 25 atoms and any other unfixed atoms in the embedded cluster have been compared as this is where significant changes in spin density and charge were calculated. f is used to denote a fixed atom in the calculation, where the geometry is unable to change during the optimisation process.

		Changes in spin density and charge compared between no U(VI) model and U(VI) incorporated	
atoms	Type and fixing	Change in spin density	Change in charge
1	Fe	0.004	-0.024
2	O f	0.000	-0.005
3	O	0.009	-0.030
4	O	-0.006	-0.015
5	H f	0.000	-0.004
6	H	0.001	-0.008
7	H	0.000	-0.001
8	O f	0.030	0.024
9	O f	0.026	-0.001
10	O f	-0.008	-0.008
11	O	0.261	-0.271
12	O	0.239	-0.299
13	O	0.251	-0.302

14	O f	-0.016	-0.013
15	O f	-0.537	-0.250
16	H f	-0.007	-0.001
17	O f	-0.216	-0.199
18	Fe	-0.019	-0.043
19	U		
20	Fe f	0.183	-0.239
21	O	0.108	-0.222
22	O	0.000	-0.027
23	H	-0.001	-0.029
24	H	0.103	-0.183
25	O	-0.025	-0.018
96	O	-0.024	0.016
97	H	0.000	-0.015
98	H	-0.002	-0.030
99	O	-0.014	0.012
100	H	0.000	-0.013
101	H	-0.001	-0.021

Table S36: Change in number of unpaired electrons and charge for each atom after addition of uranium species for when U(VI) species are incorporated at the surface without an iron vacancy in the solvated [010] goethite model. The first 25 atoms and any other unfixed atoms in the embedded cluster have been compared as this is where significant changes in spin density and charge were calculated. f is used to denote a fixed atom in the calculation, where the geometry is unable to change during the optimisation process.

		Changes in spin density and charge compared between no U(VI) model and U(VI) incorporated	
atoms	Type and fixing	Change in spin density	Change in charge
1	U		
2	O	-0.043	-0.007
3	O	0.123	-0.235
4	O	0.179	-0.278
5	H	0.000	-0.008
6	H	-0.003	-0.031
7	H	-0.003	-0.030
8	O	0.017	0.009
9	O	0.286	-0.277
10	O	0.298	-0.268
11	O	0.190	-0.053
12	O	0.006	-0.004
13	O	0.181	-0.207

14	O	0.014	0.018
15	O	-0.024	-0.014
16	H	0.000	-0.007
17	O f	-0.006	-0.005
18	Fe	0.051	-0.009
19	Fe f	0.009	0.006
20	Fe f	-0.019	-0.029
21	O f	0.009	0.003
22	O f	0.028	0.013
23	H f	0.000	-0.002
24	H f	0.000	-0.001
25	O f	0.009	0.014
97	O	0.014	-0.020
98	H	-0.001	-0.001
99	H	0.001	-0.008
100	O	0.049	-0.105
101	H	0.000	-0.024
102	H	0.001	-0.032

Section 8: List of models made with pictures.

Below is a list of all the models made and used, where any model with an incorporated uranium was studied in oxidation states 4-6. For all models, white spheres represent hydrogen atoms, red spheres represent oxygen atoms and gold spheres represent iron. For the oxygen there are two different oxygen environments, a hydroxyl environment, and a bridging oxygen environment between two iron atoms. Dashed lines are used to represent hydrogen bonding. The post-optimisation coord files for all models, in each oxidation state, can be found in the Mendeley database: Hatton, Corinne (2024), "Uranium Incorporation into [010] goethite input files", Mendeley Data, V1, doi: 10.17632/c7wczbh8w7.1

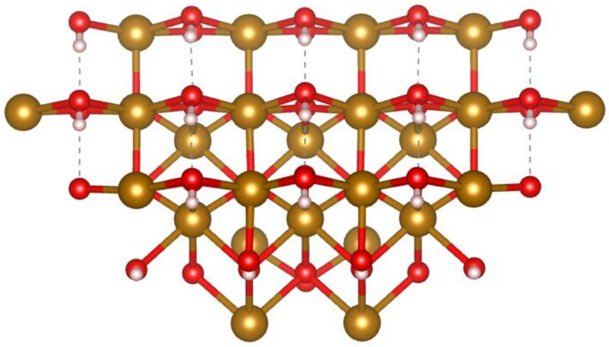

Figure S54: Model 1. Pure goethite [010] surface.

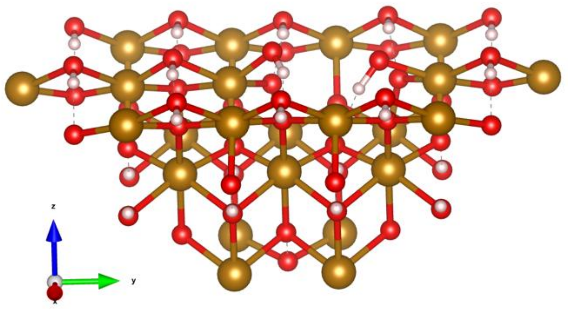

Figure S55: Model 2. Pure goethite [010] surface with a central, iron vacancy.

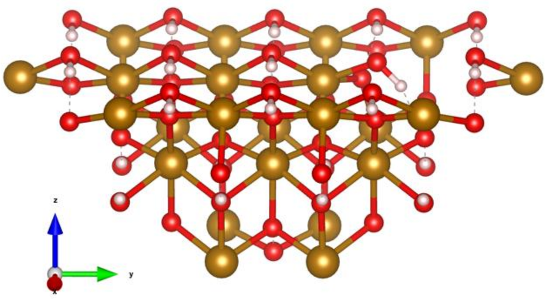

Figure S56: Model 3. Pure goethite [010] surface with a non-central iron vacancy.

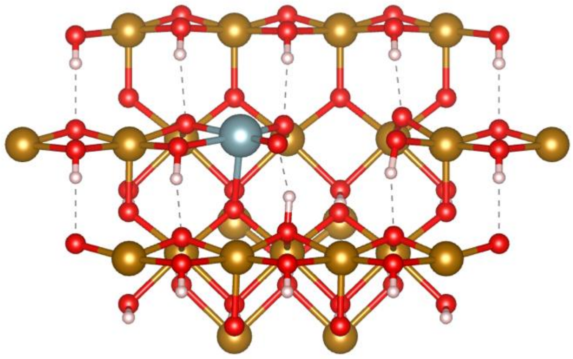

Figure S57: Model 4. Goethite [010] surface with an iron vacancy adjacent to surface incorporated U, where U was incorporated as U(IV-VI).

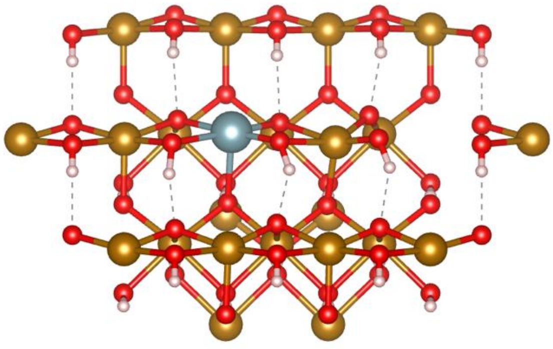

Figure S58: Model 5. Goethite [010] surface with an iron vacancy separated to surface incorporated U, where U was incorporated as U(IV-VI).

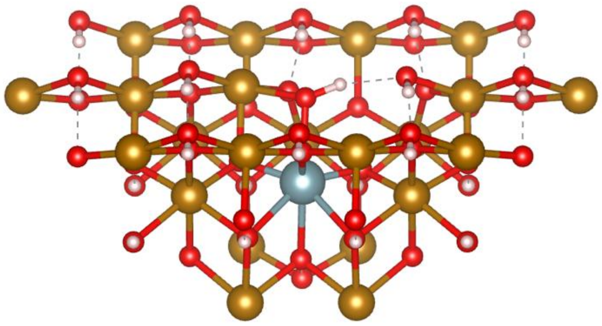

Figure S59: Model 6. Goethite [010] surface with an iron vacancy adjacent to near-surface incorporated U, where U was incorporated as U(IV-VI).

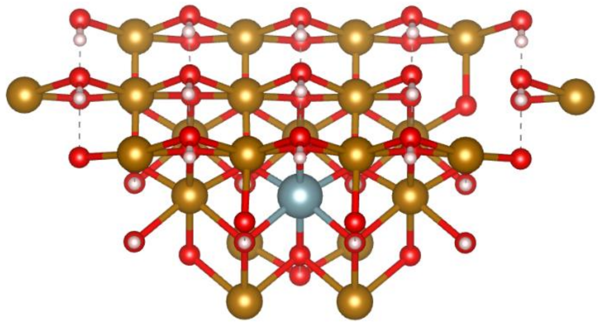

Figure S60: Model 7. Goethite [010] surface with an iron vacancy adjacent to near-surface incorporated U, where U was incorporated as U(IV-VI).

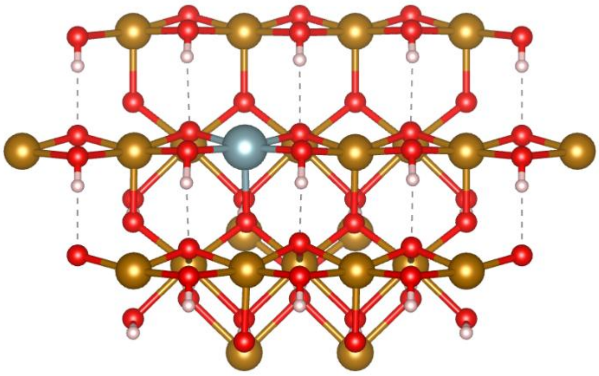

Figure S61: Model 8. Goethite [010] surface without an iron vacancy with a surface incorporated U, where U was incorporated as U(IV-VI).

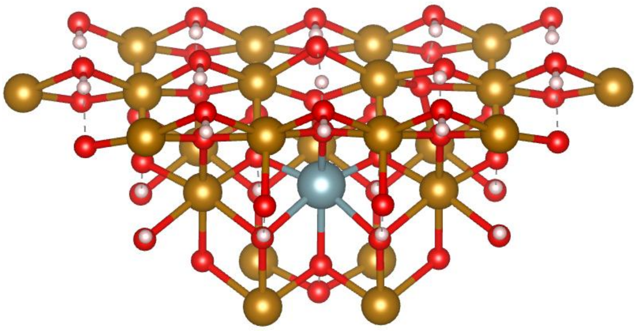

Figure S62: Model 9. Goethite [010] surface without an iron vacancy adjacent to near-surface incorporated U, where U was incorporated as U(IV-VI).

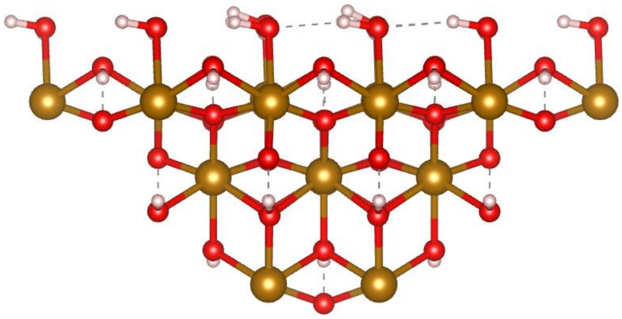

Figure S63: Model 10. Solvated pure goethite [010] surface.

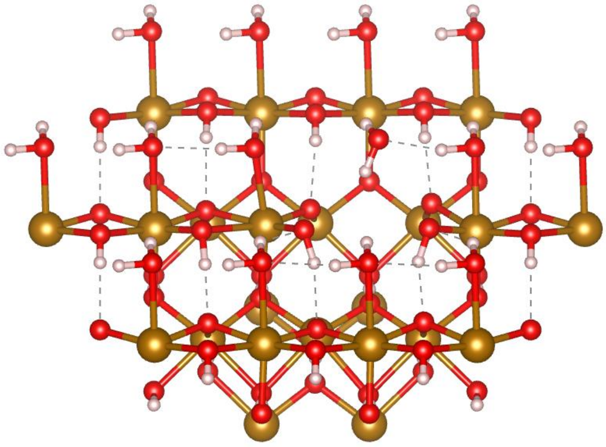

Figure S64: Model 11. Solvated pure goethite [010] surface with a central iron vacancy.

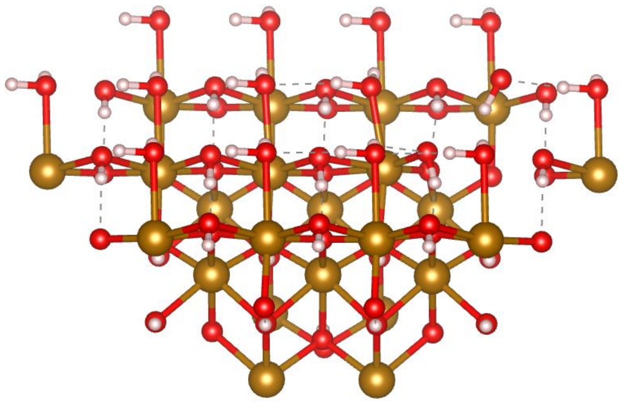

Figure S65: Model 12. Solvated pure goethite [010] surface with a non-central iron vacancy.

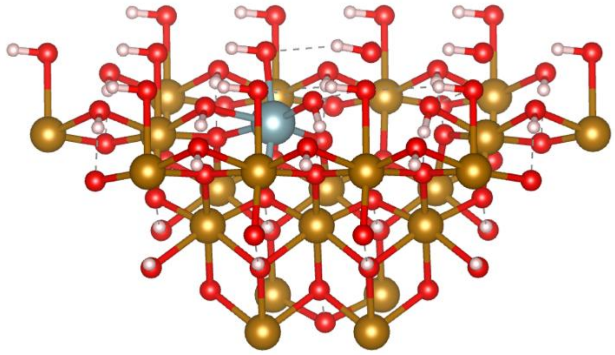

Figure S66: Model 13. Solvated goethite [010] surface with an iron vacancy adjacent to surface incorporated U, where U was incorporated as U(IV-VI).

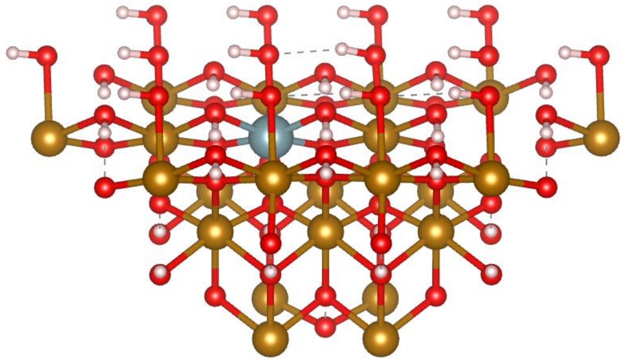

Figure S67: Model 14. Solvated goethite [010] surface with an iron vacancy separated from the surface incorporated U, where U was incorporated as U(IV-VI).

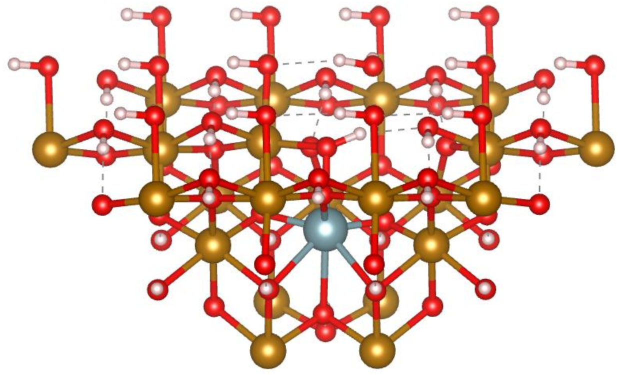

Figure S68: Model 15. Solvated goethite [010] surface with an iron vacancy adjacent to near-surface incorporated U, where U was incorporated as U(IV-VI).

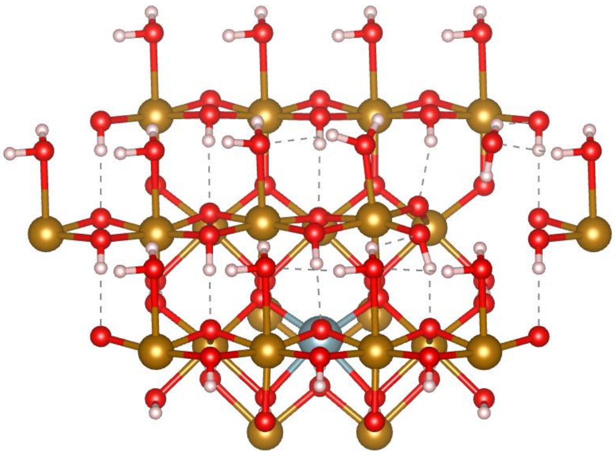

Figure S69: Model 16. Solvated goethite [010] surface with an iron vacancy separated from the near-surface incorporated U, where U was incorporated as U(IV-VI).

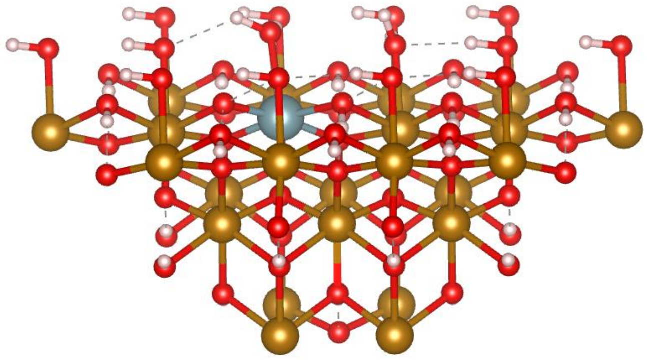

Figure S70: Model 17. Goethite [010] surface without an iron vacancy with surface incorporated U, where U was incorporated as U(IV-VI).

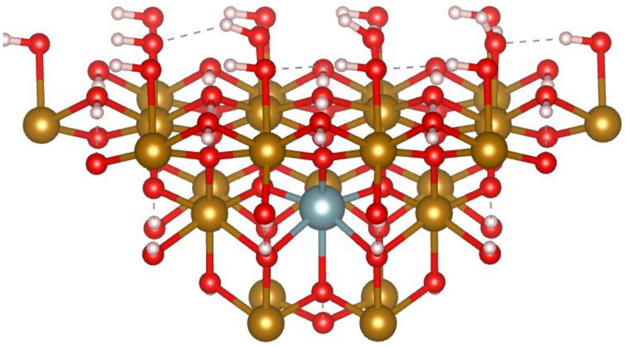

Figure S71: Model 18. Goethite [010] surface without an iron vacancy with near-surface incorporated U, where U was incorporated as U(IV-VI).
